# The chromatin remodeler CHD6 promotes colorectal cancer development by regulating TMEM65-mediated mitochondrial dynamics via EGF and Wnt signaling

**DOI:** 10.1038/s41421-022-00478-z

**Published:** 2022-12-06

**Authors:** Boyu Zhang, Qingxin Liu, Weijie Wen, Han Gao, Wenxia Wei, Aiwen Tang, Baifu Qin, Haiwen Lyu, Xiangqi Meng, Kai Li, Huilin Jin, Fenghai Yu, Qihao Pan, Junzhong Lin, Mong-Hong Lee

**Affiliations:** 1 Guangdong Provincial Key Laboratory of Colorectal and Pelvic Floor Diseases, The Sixth Affiliated Hospital, Sun Yat-sen University, Guangzhou, Guangdong China; 2 Guangdong Institute of Gastroenterology, Guangzhou, Guangdong China; 3 Department of Colorectal Surgery, Cancer Center, Sun Yat-sen University, Guangzhou, Guangdong China; 4 Department of Oncology, The Sixth Affiliated Hospital, Sun Yat-sen University, Guangzhou, Guangdong China

**Keywords:** Gastrointestinal cancer, Cell signalling

## Abstract

Chromodomain helicase DNA binding protein (CHD) family plays critical roles in regulating gene transcription. The family is linked to cancer disease, but the family member’s role in tumorigenesis remains largely unknown. Here, we report that CHD6 is highly expressed in colorectal cancer (CRC). *CHD6* knockdown inhibited cancer cell proliferation, migration, invasion, and tumorigenesis. Consistently, Villin-specific *Chd6* knockout in mice attenuates cancer formation in AOM/DSS model. We found that aberrant EGF signals promoted the stability of CHD6 by diminishing ubiquitin-mediated degradation. EGF signal inhibits GSK3β activity, which in turn prevents phosphodegron formation of CHD6, thereby hindering E3 ligase FBXW7-mediated CHD6 ubiquitination and degradation. CHD6’s chromatin remodeler activity engages in binding Wnt signaling transcription factor TCF4 to facilitate the transcriptional expression of TMEM65, a mitochondrial inner membrane protein involved in ATP production and mitochondrial dynamics. In addition, Wnt signaling is also an upstream regulator of CHD6. *CHD6* promoter contains TCF4 and β-catenin binding site, and *CHD6* can be transcriptionally activated by Wnt ligand to facilitate *TMEM65* transcription. Thus CHD6-TMEM65 axis can be regulated by both EGF and Wnt signaling pathways through two different mechanisms. We further illustrate that CHD6-TMEM65 axis is deregulated in cancer and that co-administration of Wnt inhibitor LGK974 and the anti-EGFR monoclonal antibody cetuximab largely restricted the growth of patient-derived xenografts of CRC. Targeting CHD6-TMEM65 axis may be effective for cancer intervention.

## Introduction

Colorectal cancer (CRC) is a genetic disease with accumulated genetic alterations during the progression and invasion^1^. CRC is highly aggressive and is the third leading malignance in the world population, causing near 900,000 death per year, and its incidence has been a health care challenge^2^. Despite treatment progress has been made, CRC continues to be one of the deadliest cancer types with different molecular phenotypes/strong resistance to therapies^3^ and very high mortality rate^2^. Thus, there is an urgent need to identify more molecular biomarkers for CRC. Further, it is critical to explore the molecular mechanisms underlying CRC progression, which may help provide new therapeutic targets.

Chromodomain helicase DNA-binding protein (CHD) family is composed of several members. This group of enzymes belongs to the SNF2 superfamily of ATP-dependent chromatin remodelers^4^. CHD proteins contain nine members and can be classified into three subfamilies on the basis of their functional domain similarity^4^. CHD activities are critical for a wide spectrum of cellular functions, including transcriptional regulation, cell growth, cell death, development, virus infection^5^, autophagy^6^, DNA damage response^7^, and genome integrity^4^. Deregulations of CHD in various human cancers are emerging, indicating that chromatin dynamics is critical during tumorigenesis. Subfamily III includes CHD6, CHD7, CHD8, and CHD9. These four enzymes are similar in their constituent domains, but they have non-redundant roles in the cell functions. The mechanisms behind their distinct and non-overlapping activities are unclear. Low CHD7 expression leads to enhanced survival of pancreatic cancer patients, suggesting that CHD7 promotes oncogenesis^8^. CHD8 is critical in recruiting E2F1 to the promoter of cyclin E2 for transcriptional activation^9^. The roles of CHD subfamily III proteins, including CHD6 and CHD9, in cancer remain to be characterized. *CHD9* mutations have been found in gastric and colorectal cancers^10^. Functional analysis of the large CHD6 protein (2715 aa) has been hindered by its large size.

FBXW7 is an important component of the SCF (SKP1–CUL1–F-box protein) ubiquitin E3 ligase complex^11^. FBXW7 mutations are observed in cancers^12–16^. FBXW7 is a transcriptional target of tumor suppressor p53^17,18^. FBXW7 binds target proteins and facilitates their ubiquitination and degradation^19^. Most of the FBXW7 target proteins are oncoproteins, including c-MYC^20,21^, Cyclin E^22^, c-JUN^23,24^, mTOR^25^, Notch^26^, Aurora B^27^, FOXO4, and MCL-1^24,28–32^. FBXW7 is a tumor suppressor^19^ as manifested in many types of cancers with *FBXW7* mutation^33,34^. The functional activity of FBXW7 is important in hindering cancer growth. Nonetheless, many FBXW7 targets involved in tumorigenesis remain to be characterized.

Here, we show that CHD6 is overexpressed in CRC. We have characterized the upstream regulators of the CHD6 during tumorigenesis, including EGF, glycogen synthase kinase-3β (GSK3β), FBXW7α (hereafter abbreviated as FBXW7), and Wnt3a. Moreover, we identify CHD6 downstream target TMEM65, which is a mitochondrial protein overexpressed in many cancers and is involved in regulating mitochondrial dynamics. Our results shed light on the EGF-GSK3β axis in preventing FBXW7-mediated CHD6 degradation and the Wnt-β-catenin axis in promoting the *CHD6* gene transcription during tumorigenesis, and illustrate a role of CHD6 in regulating downstream target gene *TMEM65* through Wnt signaling and TCF4 transcriptional activation. CHD6 collaborates with TCF4 to positively regulate *TMEM65* gene expression, thereby impacting mitochondrial homeostasis and promoting cancer growth and metastasis. Our understanding of the biological role of CHD6 in regulating TMEM65-mediated mitochondrial dynamics and metastasis of cancer reveals therapeutic strategies for cancer intervention and treatment.

## Results

### CHD6 is overexpressed in CRC

**Fig. 1:**
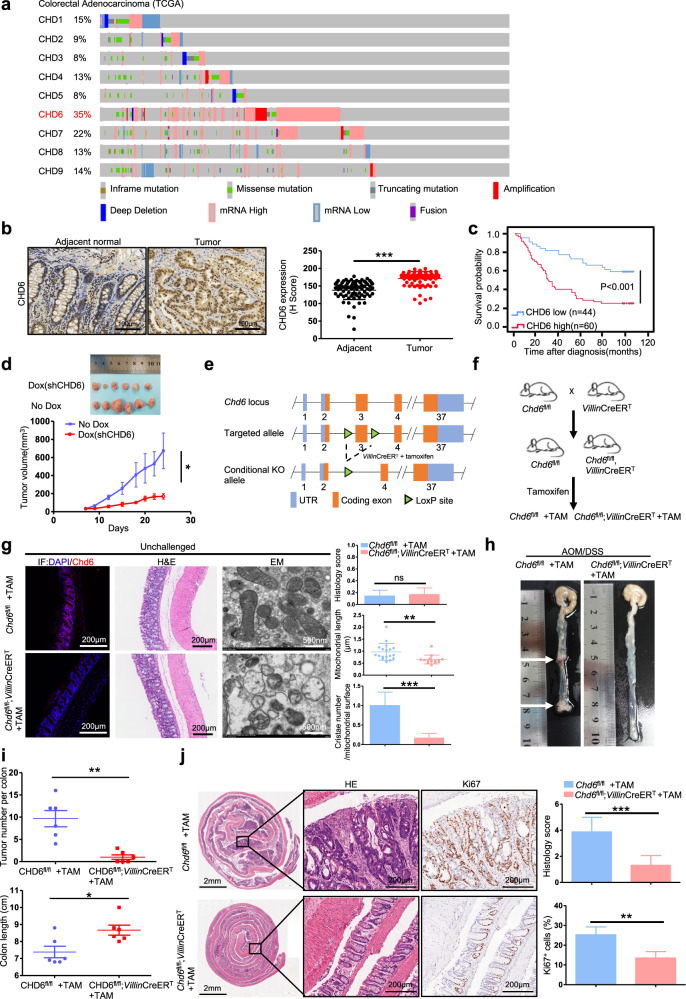
CHD6 promotes CRC tumorigenesis. **a** Genomic alterations of CHD6 and other CHD6 family members in the TCGA Colorectal Adenocarcinoma database. **b** Representative images of IHC staining for CHD6 in human colon cancer and adjacent normal colon tissue (left). A plot showing the relative expression of CHD6 in 76 paired samples of CRC and adjacent normal colon tissue (right). Scale bars, 100 μm. Data are presented as means ± SD. ***P < 0.001. *P* values were calculated by two-tailed *t*-test. **c** Kaplan–Meier survival curves of overall survival duration based on CHD6 expression in the TMA containing 104 CRC cases. The receiver operating characteristic curve was used to define the cutoff, and log-rank analysis was used to test for significance. **d** Images of subcutaneous tumors derived from HCT116 cells expressing PLKO-Tet-on-sh*CHD6*#1, with (sh*CHD6*) or without doxycycline (shCTL) treatment (top). Growth curves of subcutaneous tumors (bottom). Data are presented as means ± SD (*n* = 6 per group). *P < 0.05. *P* value was calculated by two-way ANOVA. **e** Schematic depiction of generating *Chd6* CKO mouse model. **f** Cartoon illustration of a cross between *Chd6*^fl/fl^ and *Villin-Cre*^ER^^T^ to breed *Chd6*^fl/fl^;*Villin*-*Cre*^ER^^T^ (*Chd6* CKO) mice. The *Chd6* CKO mice were induced with tamoxifen (TAM) two weeks before AOM/DSS treatment. **g** Representative images of immunofluorescence staining (IF, left), H&E staining (middle), and TEM (right) of colon tissues obtained from the indicated unchallenged mice. Histology score analysis of H&E staining and quantification of mitochondrial length and cristae number of TEM were shown as bar graph. Data are presented as means ± SD. ns, no significance, **P < 0.01. *P* values were calculated by two**-**tailed *t*-test. **h** Representative images of colons from *Chd6*^fl/fl^ + TAM (*n* = 6) and *Chd6*^fl/fl^;*Villin*-*Cre*^ER^^T^ + TAM (*n* = 6) mice treated with AOM/DSS. Arrows indicate tumors. **i** Quantification of tumor incidence per colon (top) and colon length (bottom) of *Chd6*^fl/fl^ + TAM (*n* = 6) or *Chd6*^fl/fl^;*Villin-Cre*^ER^^T^ + TAM (*n* = 6) mice with AOM/DSS treatme*n*t. Data are presented as means ± SD. *P < 0.05, **P < 0.01. *P* values were calculated by two-tailed *t*-test. **j** Representative H&E staining and Ki67 IHC staining on the colon sections obtained from the indicated mice with AOM/DSS treatment (left). Histology score analysis of H&E staining and quantification of IHC staining were shown as bar graph (right). Data are presented as means ± SD. ***P < 0.001, **P < 0.01. *P* values were calculated by two-tailed *t*-test.

To identify the potential dysregulated genes in CRC, we searched for the TCGA database and found that CHD family members were all altered in CRC. CHD6 ranked first with a 35% alteration rate (Fig. 1a). From the TCGA pan-cancer data, we also demonstrated that *CHD6* was highly amplified in CRC (Supplementary Fig. S1a). In addition, *CHD6* mRNA (encoding 2715 aa) level was higher in CRC adenocarcinoma tissues than in normal tissues (Supplementary Fig. S1b). To further investigate this phenomenon, we also detected high *CHD6* mRNA level in eighteen CRC cancer samples (Supplementary Fig. S1c). Furthermore, Kaplan–Meier analyses of the data from colon cancer dataset (GSE39582) revealed that high *CHD6* level correlated with poor relapse-free survival (Supplementary Fig. S1d). Consistent with *CHD6* mRNA upregulation, immunohistochemistry (IHC) staining of a panel of 104 CRC and 76 corresponding normal tissue specimens (tissue microarray (TMA)) showed that CHD6 protein expression is significantly higher in CRC than in adjacent normal tissue (66 of 76 paired samples (86.8%)) (Fig. 1b). Kaplan–Meier analysis curves showed that high CHD6 protein expression correlated with poor overall survival in CRC patients (Fig. 1c). Collectively, these data showed that CHD6 may play a critical role during CRC progression.

To identify the role of CHD6 in CRC, we introduced shRNA-mediated *CHD6* knockdown (KD) in DLD1, HCT116, and SW620 cells and validated the KD efficiency by western blot (Supplementary Fig. S2a). *CHD6* KD in these CRC cells inhibited/decreased cell proliferation, colony formation, cell migration, invasion, sphere formation, patient-derived organoid (PDO)^35^ growth, G1-S progression, cell survival, oxygen consumption rate (OCR), ATP production, and mitochondrial mass (FACS analysis of Mitotracker Red staining) (Supplementary Figs. S2b–e, S3a–g). Significantly, mouse subcutaneous CRC xenograft model showed that *CHD6* KD (Dox-inducible *CHD6* KD) showed a lower tumor burden (Fig. 1d; Supplementary Fig. S4a, b).

### Villin-specific CHD6 knockout attenuates cancer formation in AOM/DSS model

To further investigate whether CHD6 in intestine is involved in tumorigenesis, we established *Chd6*^fl/fl^ + TAM (tamoxifen) mice and inducible villin-specific conditional *Chd6*-knockout (*Chd6*^fl/fl^;*Villin*-*Cre*^ER^^T^ + TAM) mice (Fig. 1e, f). Correct villin Cre-mediated excision of the *Chd6* in intestine was confirmed by PCR (Supplementary Fig. S4c). We found that knockout of *Chd6* did not show any histological difference (Fig. 1g) in the colon section when compared with the control group based on hematoxylin and eosin (H&E) staining, and that knockout of *Chd6* did not show any difference in terms of goblet number as demonstrated by Alcian blue-periodic acid Schiff’s (AB-PAS) staining at three weeks after TAM injection (Supplementary Fig. S4d). However, transmission electron microscopy (TEM) analysis of mitochondria in mouse colon tissue demonstrated mitochondrial fragmentation phenotype (reduced mitochondrial length) and decreased number of cristae in *Chd6*-knockout colon cells (Fig. 1g). We then investigated the role of Chd6 in the colitis-associated CRC model. In this model, *Chd6*^fl/fl^ + TAM mice (control wild type (WT)) and *Chd6*^fl/fl^;*Villin-Cre*^ER^^T^ + TAM mice (*Chd6* conditional knockout (CKO)) were injected with Azoxymethane (AOM), followed by Dextran Sodium Sulfate (DSS) treatment (Supplementary Fig. S4e). Notably, AOM/DSS models of *Chd6* CKO mice seemed to have reduced number of tumors in the colon, a longer colon length (Fig. 1h, i), and better histology (Fig. 1j) on day 80 when compared with *Chd6*^fl/fl^ mice. *Chd6* CKO mice also tended to have less body weight loss during the DSS treatment (Supplementary Fig. S4f). Colorectal tissues from *Chd6* CKO mice exhibited a significantly reduced Ki67 staining while tumors of control WT mice have abundant Ki67 staining (Fig. 1j). Immunofluorescence staining of CHD6 further validated the deletion of *Chd6* in *Chd6* CKO mice for AOM/DSS model (Supplementary Fig. S4g). Taken together, these data indicate that CHD6 has a pivotal role in colon cancer development.

### EGF activation leads to CHD6 protein stabilization

**Fig. 2:**
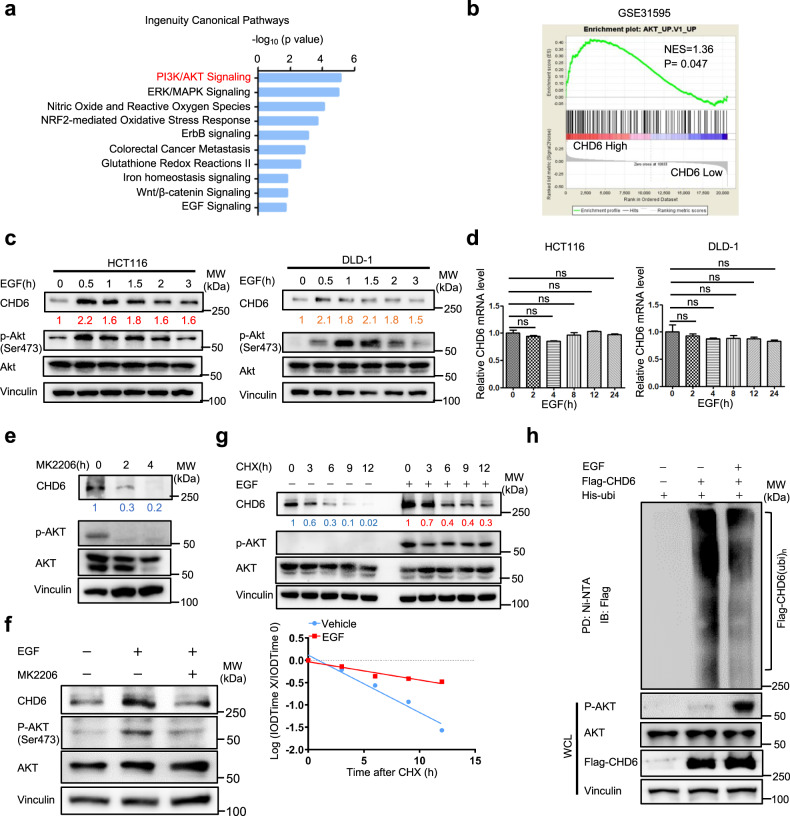
EGF increases CHD6 stability. **a** Ten cancer-related pathways that were significantly associated with genes affected by CHD6 in CRC (GSE2109 and GSE14333). Hallmark pathways emerged following IPA ‘core analysis’. Enrichment scores were displayed as −log_10_ (*P* value) by Fisher’s exact test. **b** GSEA plot of AKT signaling pathway signature correlated with CHD6 highly related genes. Normalized enrichment score (NES) and nominal *P* value of correlation were shown. **c** Representative immunoblot analysis of the indicated proteins in HCT116 and DLD-1 cells treated with EGF (100 ng/mL) at the indicated time points. **d** *CHD6* mRNA levels in the indicated cells treated with EGF (100 ng/mL) for the indicated hours. ns, not significant. **e** Representative immunoblot analysis of the indicated proteins in HCT116 cells treated with MK2206 (5 μM) at different time points. **f** Representative immunoblot analysis of the indicated proteins in HCT116 cells treated with or without MK2206, followed by EGF treatment. **g** Representative immunoblots showing CHD6 protein turnover rate in HCT116 cells treated with cycloheximide (CHX, 60 μg/mL), in the presence or absence of EGF (100 ng/mL) treatment (top). Quantification of **g** (bottom). IOD, integrated optical density. The relative density of CHD6 was normalized to Vinculin and then normalized to the *t* = 0 control. **h** Representative immunoblots showing ubiquitination of Flag-tagged CHD6, under EGF (100 ng/mL) treatment in 293T cells. Cells were treated with MG132 (10 μM) 6 h before harvest. The cell lysates were pulled down (PD) with nickel beads (Ni-NTA) and immunoblotted with the indicated antibodies. WCL whole cell lysate.

Given the high expression level and the oncogenic role of CHD6 in CRC, we sought to uncover the upstream regulators of CHD6. We analyzed two publicly available datasets (GSE2109 and GSE14333) using Ingenuity Pathway Analysis (IPA) software (Ingenuity® Systems, www.ingenuity.com). The ten most significantly enriched cancer-related pathways commonly associated with high-CHD6 group in both datasets were selected and plotted (Fig. 2a). Among these pathways, we selected PI3K/AKT and EGF signaling pathways as potential upstream regulators of CHD6 since PI3K/AKT was prominently presented in both IPA (Fig. 2a) and gene set enrichment analysis (GSEA) (Fig. 2b), and EGFR signaling is highly activated in CRC. Therefore, we investigated the dynamics of EGFR activation and CHD6 regulation. Immunoblotting analysis demonstrated that EGF treatment increased the steady-state expression of CHD6 within 0.5 h without changing its mRNA level (Fig. 2c, d). As expected, PKB/AKT was activated in response to EGF treatment (Fig. 2c), suggesting that EGF-regulated CHD6 steady-state expression may involve PKB/AKT activation. Indeed, AKT inhibitor (MK2206) treatment significantly reduced CHD6 steady-state expression with or without EGF treatment (Fig. 2e, f). We further showed that EGF treatment reduced the turnover rate of CHD6 and decreased the ubiquitination level of CHD6 (Fig. 2g, h). These data showed that EGF signaling attenuates ubiquitin-mediated degradation of CHD6, thereby increasing CHD6 protein stability.

### FBXW7 is involved in regulating CHD6 ubiquitination and degradation

**Fig. 3:**
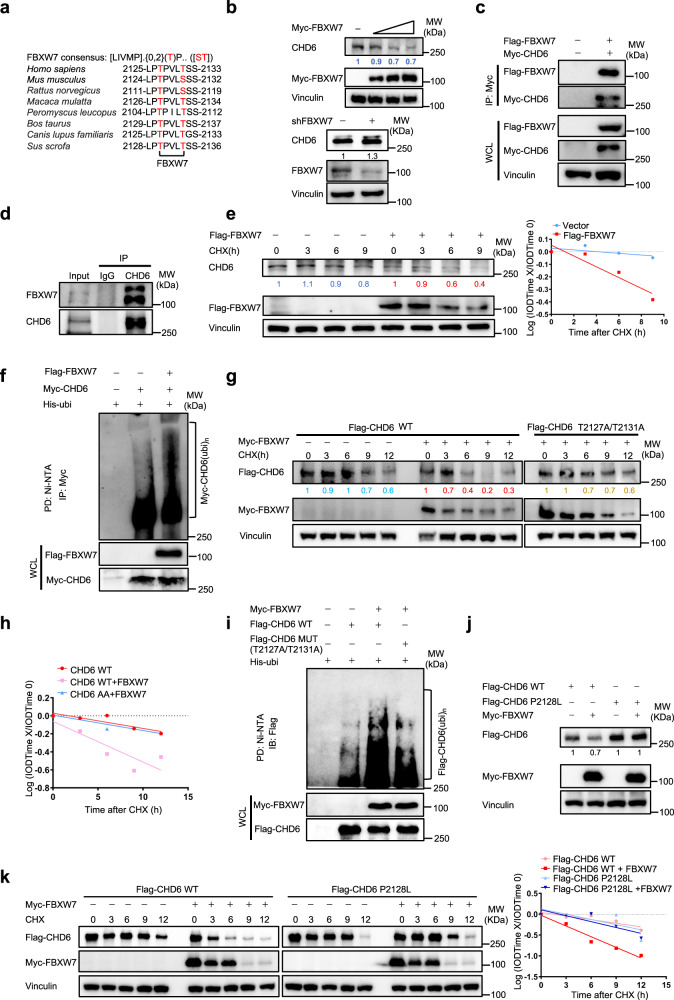
FBXW7 negatively regulates CHD6 stability. **a** Sequence alignment of the putative FBXW7-recognized degron on CHD6. [LIVMP] refers to a group of amino acids, Leu, Ile, Val, Met, Pro, are all allowed; {0,2}, 0, 1, and 2 positions of any kind of amino acids; (T), phosphorylated Thr;.., any kinds of amino acids. **b** Representative immunoblots showing CHD6 steady-state expression in HCT116 cells upon FBXW7 overexpression or KD. **c** The interaction between exogenous FBXW7 and CHD6 was determined by co-IP assay. 293T cells were transfected with Myc-tagged CHD6 and Flag-tagged FBXW7. The cell lysates were pulled down with Myc-tagged magnet beads and immunoblotted with the indicated antibodies. WCL: whole cell lysates. **d** The interaction between endogenous FBXW7 and CHD6 was determined by co-IP assay. HCT116 cell lysates were pulled down with an anti-CHD6 antibody and immunoblotted with FBXW7. **e** Representative immunoblots showing CHD6 protein turnover rate in HCT116 cells, with or without FBXW7 overexpression (left). Quantification of CHD6 turnover rate (right). Cells were transfected with the indicated plasmids in the presence of CHX (60 μg/mL) for the indicated times. CHD6 levels were normalized to Vinculin and then normalized to the *t* = 0 control. **f** 293T cells were transfected with the indicated plasmids and treated with MG132 (10 μM) 6 h before harvest. The cell lysates were pulled down with nickel beads and immunoblotted with an anti-Myc antibody. **g**, **h** Representative immunoblots showing the turnover rate of Flag-tagged CHD6 WT or Flag-tagged CHD6 T2127A/T2131A, with or without Myc-tagged FBXW7 overexpression in 293T cells (**g**). Quantification of CHD6 turnover rate (**h**). Transfected cells were treated with CHX (60 μg/mL) for the indicated times. Flag-tagged CHD6 levels were first normalized to Vinculin, and then to the *t* = 0 control. **i** 293T cells were transfected with the indicated plasmids and treated with MG132 (10 μM) 6 h before harvest. The cell lysates were pulled down with nickel beads and immunoblotted with an anti-Flag antibody. **j** 293T cells were transfected with the indicated plasmids. The cell lysates were immunoblotted with the indicated antibodies. **k** Representative immunoblots showing the turnover rate of Flag-tagged CHD6 WT or Flag-tagged CHD6 P2128L, with or without Myc-tagged FBXW7 overexpression in 293T cells (left). Transfected 293T cells were treated with CHX (60 μg/mL) for the indicated times. Quantification of CHD6 WT and P2128L mutant turnover rate (right). Flag-tagged CHD6 levels were first normalized to Vinculin, and then to the *t* = 0 control.

To determine whether a specific E3 ligase is involved in CHD6 ubiquitination, we analyzed the CHD6 peptide sequence and found that FBXW7 binding motif (2125 LPTPXXT 2131) or degron is present in CHD6 (Fig. 3a). Indeed, FBXW7 overexpression decreased the steady-state expression of CHD6 while FBXW7 KD increased the CHD6 expression (Fig. 3b). Co-immunoprecipitation (co-IP) studies indicated the exogenous and endogenous interaction between CHD6 and FBXW7 (Fig. 3c, d). Notably, FBXW7 overexpression accelerated the turnover rate of CHD6 and increased the ubiquitination of CHD6 (Fig. 3e, f).

To confirm that FBXW7 targeted the specific binding motif (LPTPXXT) or phosphodegron for CHD6 degradation, we constructed the CHD6 T2127A/T2131A mutant within (2125 LPTPXXT 2131) motif. The results showed that FBXW7 accelerated the turnover rate of CHD6 WT but not the CHD6 T2127A/T2131A mutant (Fig. 3g, h). Again, CHD6 WT is vulnerable to FBXW7-mediated ubiquitination, while CHD6 T2127A/T2131A mutant is resistant to FBXW7’s impact (Fig. 3i). Based on the CRC genome sequence data, we identified a cancer-derived mutation (P2128L) in CHD6 within the binding motif. We then constructed the patient-derived CHD6 mutant (P2128L) and demonstrated that cancer-derived CHD6 P2128L is resistant to FBXW7-mediated degradation and turnover (Fig. 3j, k). Together, these results demonstrated that FBXW7-mediated downregulation of CHD6 requires the phosphorylation of FBXW7 motif on CHD6.

### Tumor suppressor FBXW7 promotes CHD6 ubiquitination through binding phosphodegron of CHD6 in a GSK3β-dependent manner

**Fig. 4:**
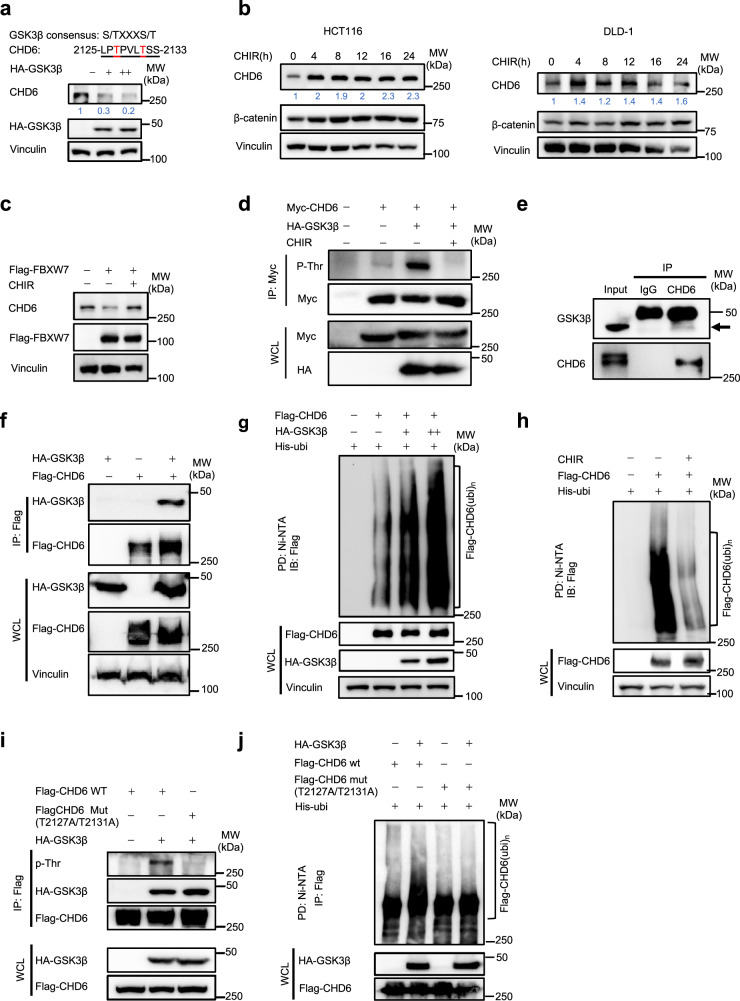
FBXW7 promotes CHD6 ubiquitination in a GSK3β-dependent way. **a** Consensus sequence of the putative GSK3β phosphorylation motif of CHD6 (T2127 and T2131) (top). Representative immunoblots showing CHD6 steady-state expression in HCT116 cells with increased GSK3β overexpression (bottom). **b** Representative immunoblots showing CHD6 steady-state expression in HCT116 and DLD-1 cells treated with GSK3 inhibitor CHIR-99021 (2 μM) at the indicated time points. **c** Representative immunoblots showing CHD6 steady-state expression in HCT116 cells transfected with the indicated plasmid in the presence of CHIR-99021 (2 μM). **d** 293T cells transfected with the indicated plasmids, in the presence or absence of CHIR-99021 (2 μM) treatment, were immunoprecipitated with an anti-Myc antibody and immunoblotted with the indicated antibodies. Threonine-phosphorylated CHD6 was shown. WCL: whole cell lysates. **e** HCT116 cell lysates were immunoprecipitated (IP) with an anti-CHD6 antibody and immunoblotted with anti-GSK3β antibody. **f** 293T cells transfected with the indicated plasmids were immunoprecipitated with an anti-Flag antibody and immunoblotted with the indicated antibodies. **g** 293T cells were transfected with the indicated plasmids and treated with MG132 (10 μM) 6 h before harvest. The cell lysates were pulled down with nickel beads and immunoblotted with the indicated antibodies. **h** 293T cells were transfected with the indicated plasmids, in the presence or absence of CHIR-99021 treatment. All plates were added with MG132 (10 μM) 6 h before harvest. The cell lysates were pulled down with nickel beads and immunoblotted with an anti-Flag antibody. **i** 293T cells were transfected with the indicated plasmids. The cell lysates were immunoprecipitated with an anti-Flag antibody and immunoblotted with the indicated antibodies. **j** 293T cells were transfected with the indicated plasmids and treated with MG132 (10 μM) 6 h before harvest. The cell lysates were pulled down with nickel beads and immunoblotted with the indicated antibodies.

Given that FBXW7 recognizes phosphorylated degron of target proteins for degradation, and the FBXW7 binding motif on CHD6 is a GSK3β-recognized motif (S/TXXXS/T) and phosphorylation site (Fig. 4a), we hypothesized that FBXW7-mediated CHD6 degradation depends on GSK3β-catalyzed phosphorylation. Indeed, CHD6 steady-state expression decreased with increasing amount of GSK3β (Fig. 4a), while GSK3 inhibitor (CHIR-99021) treatment seemed to increase the level of CHD6 (Fig. 4b). Moreover, FBXW7-mediated CHD6 downregulation was antagonized by CHIR-99021 treatment (Fig. 4c). The threonine phosphorylation level of CHD6 was increased in the presence of GSK3β, but was attenuated by CHIR-99021 treatment (Fig. 4d). Both endogenous and exogenous co-IP assays confirmed the interaction between CHD6 and GSK3β (Fig. 4e, f). More importantly, the presence of GSK3β can facilitate the ubiquitination of CHD6 (Fig. 4g). Congruently, CHIR-99021 treatment to inhibit GSK3β decreased the ubiquitination of CHD6 (Fig. 4h). To further confirm that GSK3β is involved in regulating CHD6 phosphorylation, we investigated the phosphorylation level of CHD6 (T2127A/T2131A) mutant in the presence of GSK3β. As expected, WT CHD6 threonine phosphorylation was enhanced by GSK3β. However, the CHD6 (T2127A/T2131A) mutant was resistant to GSK3β’s activity in terms of Thr phosphorylation (Fig. 4i), and thus was less vulnerable to GSK3β-mediated ubiquitination (Fig. 4j). Together, these results demonstrated that GSK3β-mediated downregulation of CHD6 requires the phosphorylation of CHD6 on phosphodegron motif (T2127/T2131).

### CHD6 transcriptionally regulates the expression of TMEM65, a marker overexpressed in cancer

**Fig. 5:**
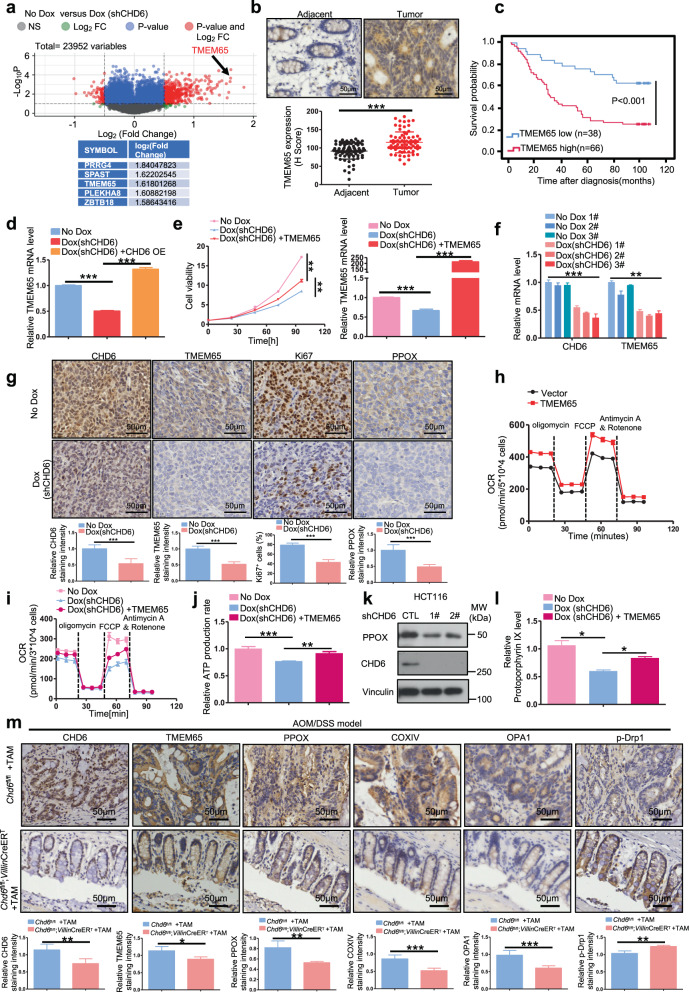
CHD6 regulates mitochondrial function through TMEM65. **a** Volcano plot generated from transcriptomic analyses of shCTL (no Dox) and sh*CHD6* (Dox) HCT116 cells. Dox, doxycycline. **b** Representative images of IHC staining for TMEM65 in human colon cancer and adjacent normal colon tissue (top). A plot showing the relative expression of TMEM65 in 76 paired samples of CRC and adjacent normal colon tissue (bottom). Scale bars, 50 μm. **c** Kaplan-Meier survival curves of overall survival duration based on TMEM65 protein expression in the 104 CRC patient tissues. The receiver operating characteristic curve was used to define the cutoff, and log-rank analysis was used to test for significance. **d** RT-qPCR analysis of *TMEM65* in HCT116 cells expressing *CHD6* shRNA with or without Flag-tagged CHD6 overexpression. **e** Growth curves of *CHD6* KD HCT116 cells in the presence of TMEM65 overexpression (left); RT-qPCR analysis confirmed the efficiency of *TMEM65* overexpression (right). **f** RT-qPCR analysis of *CHD6* and *TMEM65* in *CHD6* KD HCT116 subcutaneous tumors from Fig. 1d. **g** Representative images of IHC staining for CHD6, TMEM65, Ki67, and PPOX in subcutaneous tumor sections from Fig. 1d (top) and quantification of IHC staining by ImageJ (bottom). **h** OCR of HCT116 cells with TMEM65 overexpression. **i** OCR of shCTL and sh*CHD6* HCT116 cells, with or without TMEM65 overexpression. **j** Quantification of cellular ATP in shCTL and sh*CHD6* HCT116 cells, with or without TMEM65 overexpression. **k** Immunoblot analysis of PPOX expression in shCTL and sh*CHD6* HCT116 cells. **l** Heme levels in control and *CHD6* KD HCT116 cells, with or without TMEM65 overexpression. **m** Representative images of IHC staining for CHD6, TMEM65, PPOX, COXIV, OPA1, and p-Drp1 (Ser616) in the colon tissues obtained from the indicated mice with AOM/DSS treatment. Quantification of IHC staining shown at the bottom (samples from at least 3–6 mice for each genotype were analyzed). Assays were performed with three replicates. *P* values were calculated by two-tailed *t*-test (**b**, **g**, **m**), one-way ANOVA (**d**–**f**, **j**, **l**), or two-way ANOVA (**e**). Data are presented as means ± SD. ***P < 0.001, **P < 0.01, *P < 0.05, ns, not significant.

To identify the downstream signals of CHD6, we established Tet-on-inducible-sh*CHD6* construct for microarray analysis. The differentially expressed genes were shown in the volcano plot between the control (no Dox) and *CHD6* KD cells (Dox) (Fig. 5a). The top five downregulated genes in *CHD6* KD group were *PRRG4, SPAST*, *TMEM65*, *PLEKHA8*, and *ZBTB18*. To determine these potential downstream targets that may mediate the oncogenic effect of CHD6 in CRC, we performed correlation analysis with TCGA dataset and found that *TMEM65* mRNA expression was significantly associated with *CHD6* (Supplementary Fig. S5a); moreover, survival analyses showed that high transcriptional activation of *TMEM65* (Supplementary Fig. S5b), but not other four genes, was associated with worse prognosis for overall survival in CRC patients. Consistent with the mRNA expression profiles, IHC analyses in our cohort of CRC patients also revealed that TMEM65 protein expression was significantly increased in CRC specimens compared with that in normal adjacent tissues (Fig. 5b). Further, high expression of TMEM65 protein was associated with poor prognosis for overall survival (Fig. 5c). These analyses indicate that TMEM65 may have an oncogenic effect during CRC development. The positive regulation of TMEM65 by CHD6 was further confirmed by RT-qPCR and western blot. Notably, CHD6 overexpression increased TMEM65 mRNA and protein levels, while *CHD6* KD decreased TMEM65 mRNA and protein levels (Supplementary Fig. S5c, d). More importantly, CHD6 re-expression restored the expression of *TMEM65* mRNA in *CHD6* KD cells (Fig. 5d). Interestingly, TMEM65 can promote cancer cell growth (Supplementary Fig. S5e). To investigate whether the CHD6-TMEM65 axis plays a critical role in promoting tumorigenesis, we performed the following experiments. First, *CHD6* KD led to inhibition of cell growth, but TMEM65 expression reversed, at least in part, this impact caused by *CHD6* KD (Fig. 5e). Second, in the mouse xenograft cancer samples from Fig. 1d, *TMEM65* mRNA levels were decreased in *CHD6* KD tumors, as detected by RT-qPCR (Fig. 5f). Third, *CHD6* KD tumors from Fig. 1d contained low levels of TMEM65 with concurrent lower Ki67 signals while control tumors showed relatively higher levels of TMEM65 and higher Ki67 signals based on IHC staining (Fig. 5g), suggesting that CHD6 and TMEM65 are positively correlated. Together, the correlation between CHD6 and TMEM65 could be recapitulated in mouse xenograft cancer model, and deregulation of TMEM65 level may play roles in CHD6-mediated tumorigenicity.

### CHD6-TMEM65 axis regulates mitochondrial fusion process, OCR, and heme formation

TMEM65 is a mitochondrial inner membrane protein, and *CHD6* KD inhibits OCR and ATP production (Supplementary Fig. S3e, f). We then sought to elucidate the function of CHD6-TMEM65 axis on mitochondrial homeostasis in CRC. TMEM65 overexpression led to increased OCR (Fig. 5h). Interestingly, TMEM65 expression rescued, at least in part, the reduced OCR and low ATP production caused by *CHD6* KD (Fig. 5i, j). Further, *CHD6* KD led to reduction of protoporphyrinogen oxidase (PPOX), a protein involved in heme synthesis (Fig. 5k, g). Heme regulates cytochrome c oxidase (COX) biogenesis, which plays essential roles in oxidative phosphorylation and ATP production. As a result, *CHD6* KD led to a decrease of heme (measured after conversion to the autofluorescent precursor protoporphyrin IX (PPIX)) (Fig. 5l; Supplementary Fig. S5f). Again, TMEM65 increased heme level (Supplementary Fig. S5g), and its expression could also rescue, at least in part, the reduced heme production caused by *CHD6* KD (Fig. 5l), suggesting that CHD6-TMEM65 axis increased oxidative metabolism. Furthermore, TMEM65 expression increased PPOX while TMEM65 KD decreased PPOX (Supplementary Fig. S5h). Co-IP result showed that TMEM65 can interact with PPOX (Supplementary Fig. S5i), suggesting that TMEM65 regulates PPOX through protein–protein interaction.

Given that mitochondrial protein TMEM65 impacts mitochondrial functions and interacts with PPOX, this observation raises an interesting issue regarding the association of TMEM65 with other mitochondrial proteins in the complex. Our gel filtration studies indicate that TMEM65 are present in complexes with different mitochondrial proteins, such as ATP5a, Mt-CO2, NDUFA8, ATP5b, and PPOX, reiterating its role in regulating mitochondrial homeostasis (Supplementary Fig. S5j). Together, CHD6 regulates mitochondrial functions through TMEM65. *CHD6* KD leads to decreased TMEM65 (a PPOX interacting protein) and decreased oxidative phosphorylation and ATP production.

As mitochondrial morphology change was observed in *Chd6*-knockout mouse model (Fig. 1g), we then further examined mitochondrial dynamics and the expression of proteins involved in mitochondrial functions in *CHD6* KD cells. Confocal microscopy demonstrated the irregular and shorter mitochondrial length observed in *CHD6* KD cells (Supplementary Fig. S5k). Interestingly, expressing TMEM65 in *CHD6* KD cells can reverse this phenomenon (Supplementary Fig. S6a); namely, cells with overexpression of TMEM65 showed longer mitochondria and increased mitochondrial mass even under *CHD6* KD. Confocal microscopy showed the colocalization of TMEM65-GFP and mitochondria Mitotracker (Supplementary Fig. S6b), suggesting that TMEM65 may execute its rescue effect physically at the mitochondrial site. Consistent with the confocal picture, the morphology recorded by TEM demonstrated that *CHD6* KD cells tended to have smaller mitochondria (possibly due to fission) (Supplementary Fig. S6c). Drp1 can be recruited to mitochondrial constriction site and assembled into higher-order oligomers to facilitate fission of mitochondria. We found that *CHD6* KD led to increased Drp1 in the mitochondria, decreased COX-IV, and decreased mitochondrial transcription factor A (TFAM, an important protein interacting with mtDNA to be involved in transcription of mtDNA) (Supplementary Fig. S6d). This observation suggests that *CHD6* KD has facilitated mitochondrial fission and perturbed the mitochondrial homeostasis.

Interestingly, TMEM65 expression led to longer fused mitochondria based on TEM images (Supplementary Fig. S6e). We found that TMEM65 expression led to the reduced p-Drp1 (Ser616), increased VDAC1, and reduced Parkin (an E3 ligase involved in mitophagy), indicating its role in mitochondrial fusion and mitophagy (Supplementary Fig. S6f). Further, TMEM65 expression led to more cristae per mitochondrion (Supplementary Fig. S6g). Consistent with the in vitro findings, we also detected that TMEM65, PPOX, and VDAC1 levels were reduced, whereas p-Drp1 level was increased in *Chd6*-knockout mice compared to control *Chd6* ^*fl/fl*^ mice as demonstrated by immunoblotting (Supplementary Fig. S6h).

To examine whether the effect of CHD6-TMEM65 axis on mitochondrial function contributes to the development of colitis-associated neoplasia in the AOM/DSS model, we examined mitochondrial protein expression with IHC staining on colon tumor sections obtained from AOM/DSS-treated *Chd6* ^*fl/fl*^ and *Chd6*-knockout mice. In line with observations of unchallenged *Chd6*-knockout mice, the mitochondrial deregulations again were also observed in AOM/DSS-treated *Chd6*-knockout mouse model (Fig. 5m), including reduced levels of TMEM65, PPOX, COX-IV, optic atrophy 1 (OPA1) and increased p-Drp1 level, suggesting that loss of impact of CHD6-TMEM65 axis on mitochondrial functions plays roles in alleviating tumor formation. In conclusion, the CHD6-TMEM65 axis is critical in the regulation of mitochondrial homeostasis during the AOM/DSS-induced carcinogenesis.

### High CHD6 expression level in CRC promotes metastasis and tumorigenesis

**Fig. 6:**
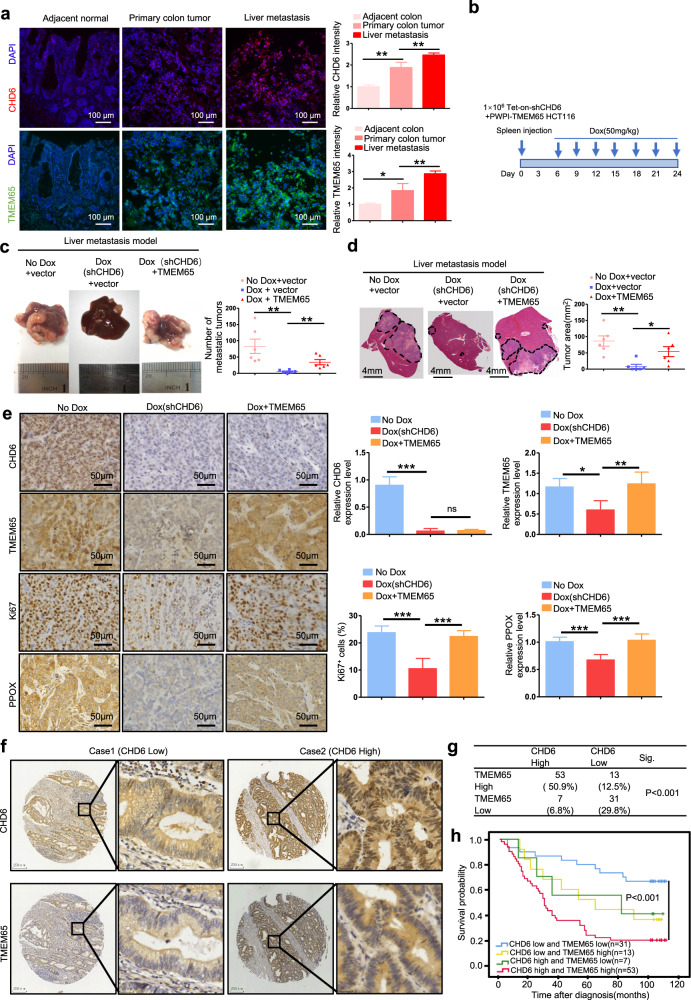
CHD6-TMEM65 axis contributes to CRC progression. **a** Representative images of immunofluorescence staining for CHD6 and TMEM65 in CRC tissue of primary site, adjacent normal tissue, and liver metastasis tissue obtained from the same CRC patient (left). Red, CHD6; green, TMEM65; blue, DAPI. The staining intensity of CHD6 and TMEM65 was quantitated by ImageJ and presented as bar graphs (right). Data are presented as means ± SD. *P* values were calculated by one-way ANOVA. *P < 0.05, **P < 0.01. **b** Schematic diagram of the establishment of liver metastasis mouse model. Liver metastasis model was established by injection of HCT116 cells into the spleen of nude mice (no Dox, *n* = 6; Dox (sh*CHD6*), *n* = 6; Dox+TMEM65 OE, *n* = 6). Dox, doxycycli*n*e. **c** Representative images of mouse liver tissue with metastatic tumors (left). Quantification of macroscopic liver metastases 4 weeks post inoculation (right). Data are presented as means ± SD. **P < 0.01; *P* values were calculated by one-way ANOVA. **d** Representative images of H&E staining on liver tissue sections (left) and quantification of metastatic tumor areas (right). The black dashed lines indicate the tumor borders; data are presented as means ± SD. *P < 0.05, **P < 0.01. *P* values were calculated by one-way ANOVA. **e** Representative images of IHC staining for CHD6, TMEM65, Ki67, and PPOX on liver metastasis sections (*n* = 6). Quantifications of IHC staining were shown as bar graphs. Data are presented as means ± SD. *P < 0.05, **P < 0.01, ***P < 0.001. *P* values were calculated by one-way ANOVA. **f** Representative images of IHC staining for CHD6 and TMEM65 in serial sections of patient TMAs that consist of 104 CRC cases. Case 1 is representative of a patient with CHD6 low-expressed colon cancer. Case 2 is representative of a patient with CHD6 high-expressed colon cancer. **g** χ^2^ analysis shows the correlation of CHD6 and TMEM65 expression in human CRC TMA specimens. **h** Kaplan–Meier survival curves of overall survival duration based on CHD6 and TMEM65 expression from TMA data. The receiver operating characteristic curve was used to define the cutoff, and log-rank analysis was used to test for significance.

Liver metastasis is commonly observed in CRC and is responsible for the high rate of mortality and morbidity in CRC patients. Many studies have shown that mitochondrial function is important for supporting cancer cell growth in the distant organs^36–38^. Indeed, in CRC patients with liver metastasis, metastatic liver cancer samples exhibited high levels of CHD6 and TMEM65 compared to CRC samples of primary site and its adjacent normal tissue sample (Fig. 6a). Thus, we hypothesized that disruption of CHD6-TMEM65 axis can impair the capacity of cancer cell to colonize the liver. To investigate our hypothesis, we established a CRC liver metastasis model by intra-splenic inoculation of Tet-on-inducible-sh*CHD6* HCT116 cells (1 × 10^6^ cells per mouse) with or without ectopic expression of TMEM65 (Fig. 6b). The data showed that *CHD6* KD led to reduced liver metastasis, which can be reversed, at least in part, by TMEM65 overexpression in terms of number of metastasis and tumor area (Fig. 6c, d), suggesting a role of CHD6-TMEM65 axis in facilitating metastasis. IHC staining on metastasized liver showed that *CHD6* KD led to decreased metastatic cell proliferation (Ki67 staining), compromised expression of TMEM65, and reduced PPOX expression. Importantly, TMEM65 overexpression reversed, at least in part, these impacts from *CHD6* KD (Fig. 6e). These results indicate that CHD6 and TMEM65 could be prognostic markers for CRC metastasis. We also validated the correlation of CHD6 and TMEM65 in 104 CRC patients by IHC of TMA and found that CHD6 and TMEM65 showed a significantly positive correlation (Fig. 6f, g). Kaplan–Meier analysis showed that the CHD6-high and TMEM65-high group tended to exhibit the poor overall survival compared to the CHD6-low and TMEM65-low group (Fig. 6h). Meanwhile, the analysis of clinical characteristics of CRC patients showed that high TMEM65 expression was positively correlated with pN status of CRC patients (Supplementary Table S1). Furthermore, multivariate Cox regression analysis revealed that TMEM65 expression is an independent prognostic factor for poor survival (Supplementary Table S2).

### CHD6 collaborates with TCF4 to transcriptionally regulate TMEM65

**Fig. 7:**
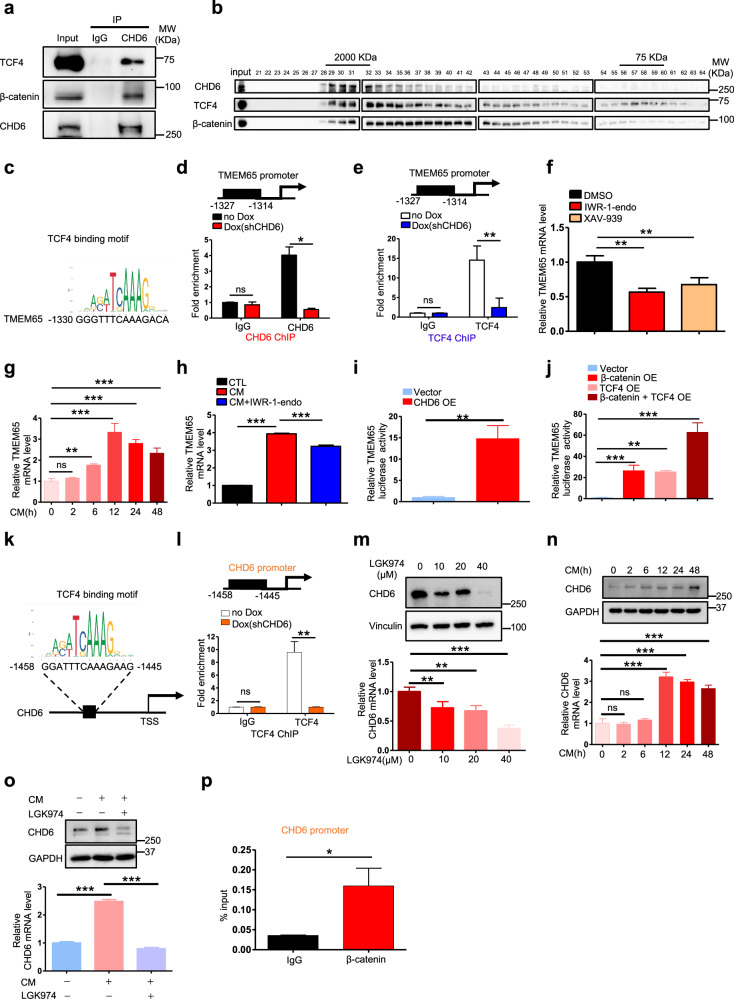
CHD6 transcriptionally regulates *TMEM65* by cooperating with TCF4. **a** HCT116 cell lysates were immunoprecipitated with an anti-CHD6 antibody and were immunoblotted with the indicated antibodies. **b** Representative immunoblot analysis of fractions containing protein complex eluted from size exclusion chromatography. Cell lysate extracted from CHD6-transfected HEK293T cells was analyzed for CHD6-containing complexes using Superose 6 (GE) gel filtration column. Eluted fractions of protein complex were collected and revealed by immunoblotting with the indicated antibodies. CHD6, TCF4, and β-catenin were co-eluted at fractions 29–32. **c** TCF4 binding motif obtained from JASPAR (top). The potential binding site of TCF4 on the promoter of *TMEM65* (bottom). **d** ChIP of CHD6 and IgG in control or *CHD6* KD HCT116 cells, followed by qPCR for the loci identified by searching for binding sites on *TMEM65* promoter. **e** ChIP of TCF4 and IgG in control and *CHD6* KD HCT116 cells followed by qPCR for the loci of predicted binding sites on *TMEM65* promoter. **f** RT-qPCR analysis of *TMEM65* mRNA in HCT116 cells treated with Wnt pathway inhibitors IWR-1-endo (20 μM), XAV-939 (1 μM), or vehicle. **g** RT-qPCR analysis of *TMEM65* mRNA in HCT116 cells cultured with L-Wnt3a-expressing cell CM. **h** RT-qPCR analysis of *TMEM65* mRNA in HCT116 cells cultured with L-Wnt3a-expressing cell CM, in the presence or absence of Wnt pathway inhibitor IWR-1-endo (20 μM). **i** *TMEM65*-reporter luciferase activity in 293T cells with or without CHD6 overexpression. **j** *TMEM65*-reporter luciferase activity in 293T cells with overexpression of β-catenin and TCF4 alone, or in combination. **k** TCF4 binding motif obtained from JASPAR (top). The potential binding site of TCF4 on the promoter of *CHD6* (bottom). **l** ChIP of TCF4 and IgG in control or *CHD6* KD HCT116 cells, followed by qPCR for the loci on *CHD6* promoter. **m** Immunoblot and RT-qPCR analysis of CHD6 in HCT116 cells treated with Wnt pathway inhibitors LGK974. **n** Immunoblot and RT-qPCR analysis of CHD6 in HCT116 cells cultured with L-Wnt3a-expressing cell CM. **o** Immunoblot and RT-qPCR analysis of CHD6 in HCT116 cells cultured with L-Wnt3a-expressing cell CM, in the presence or absence of Wnt pathway inhibitors LGK974 (10 μM). **p** ChIP of β-catenin and IgG in HCT116 cells, followed by qPCR for the loci on *CHD6* promoter. Assays were performed with three replicates. *P* values were calculated by two-tailed *t*-test (**d**, **e**, **i**, **l**, **p**) or one-way ANOVA (**f**–**h**, **j**, **m**–**o**). Data are presented as means ± SD. ***P < 0.001, **P < 0.01, *P < 0.05, ns, not significant.

Next, we tried to elucidate the underlying mechanisms by which CHD6 regulates TMEM65 expression. As a chromatin remodeler, CHD6 regulates gene transcription by increasing chromatin accessibility for transcription factors and the general transcription machinery^39^. We sought to identify the transcription factor that is involved in *TMEM65* transcription. GSEA results showed that the expression of Wnt pathway genes was positively correlated with *CHD6* and *TMEM65* expression (Supplementary Fig. S7a, b). Our array data also showed that Wnt is the most downregulated hallmark pathway in *CHD6* KD cells (Supplementary Fig. S7c), suggesting that Wnt pathway is critical for CHD6 signaling. Intriguingly, co-IP results showed that CHD6 interacted with both TCF4 and β-catenin (Fig. 7a). Moreover, gel filtration studies indicate that CHD6 are present in complexes (~2000 Kd) with TCF4 and β-catenin (Fig. 7b), suggesting that its chromatin remodeling role may be involved in regulating Wnt/ β-catenin signaling. By searching for the TCF4 consensus sequence, we found that *TMEM65* promoter contains the TCF4 binding site located between −1327 and −1314 (Fig. 7c). We performed CHD6 chromatin immunoprecipitation (ChIP) analysis and found that CHD6 bound to this TCF4 binding site (−1327 to −1314) on *TMEM65* promoter while *CHD6* KD diminished the binding (Fig. 7d). We also performed TCF4 ChIP analysis to confirm TCF4 binding to this sequence of *TMEM65* promoter (Fig. 7e). Importantly, *CHD6* KD led to reduced binding of TCF4 to this sequence on *TMEM65* promoter (Fig. 7e), suggesting that CHD6 is facilitating TCF4 binding to *TMEM65* promoter.

To further demonstrate the impact of Wnt signaling on regulating *TMEM65* gene expression, we employed Wnt pathway inhibitors (IWR-1-endo and XAV-939) and showed that they can downregulate mRNA expression of *TMEM65* (Fig. 7f). On the contrary, conditioned medium (CM) containing L-Wnt3a led to the elevation of *TMEM65* gene expression (Fig. 7g). Congruently, L-Wnt3a-mediated gene elevation of *TMEM65* can be attenuated by the presence of Wnt pathway inhibitor IWR-1-endo (Fig. 7h). Together, Wnt signaling may enhance the binding between CHD6 and TCF4 to facilitate TCF4 binding to *TMEM65* promoter, thereby strengthening transcriptional expression of *TMEM65*. Further, we have performed a *TMEM65* luciferase reporter gene assay to show that CHD6 can transcriptionally activate *TMEM65* promoter (Fig. 7i). Consistently, β-catenin and TCF4 collaborate to transcriptionally activate *TMEM65* promoter efficiently (Fig. 7j) based on a *TMEM65* luciferase reporter gene assay. Domain mapping assay demonstrated that ATPase/Helicase domain of CHD6 is required for CHD6 interaction with TCF4, illustrating the physical interaction of these two proteins (Supplementary Fig. S7d, e). Detailed studies indicated that ATPase domain of CHD6 is critical for binding TCF4 (Supplementary Fig. S7f, g). Thus, CHD6 ATPase domain physically associates with TCF4 to transcriptionally regulate *TMEM65*.

Interestingly, we also found that *CHD6* promoter contains the TCF4 binding site located between −1458 and −1445 (Fig. 7k). TCF4 ChIP analysis demonstrated that TCF4 bound to this binding site (−1458 to −1445) on *CHD6* promoter while *CHD6* KD diminished the TCF4 binding (Fig. 7l). Surprisingly, we also found that Wnt pathway inhibitors (LGK974, IWR-1-endo, and XAV939) downregulated *CHD6* expression (Fig. 7m; Supplementary Fig. S7h) while CM containing L-Wnt3a elevated *CHD6* expression (Fig. 7n). Consistently, the positive impact of CM on *CHD6* gene expression can be antagonized by Wnt inhibitors including LGK974, IWR-1-endo, and XAV939 (Fig. 7o; Supplementary Fig. S7i). β-catenin ChIP analysis indicated that β-catenin also bound to *CHD6* promoter (Fig. 7p). These results indicate that Wnt signaling may enhance the transcription of *CHD6* through TCF4/β-catenin’s transcriptional activity. This could explain, at least in part, the upregulation of *CHD6* mRNA level detected in CRC patient samples since Wnt signaling is highly deregulated in CRC. Together, CHD6 regulates *TMEM65* transcriptional expression through enhancing TCF4/β-catenin’s activity via Wnt signaling. *CHD6* gene itself is also a transcriptional target of TCF4/β-catenin in response to Wnt activity.

### Combination treatment of Cetuximab and Wnt inhibitor mitigated tumor growth in CHD6-high human patient-derived xenograft (PDX) CRC model with better efficacy through hindering CHD6-TMEM65 signaling axis

**Fig. 8:**
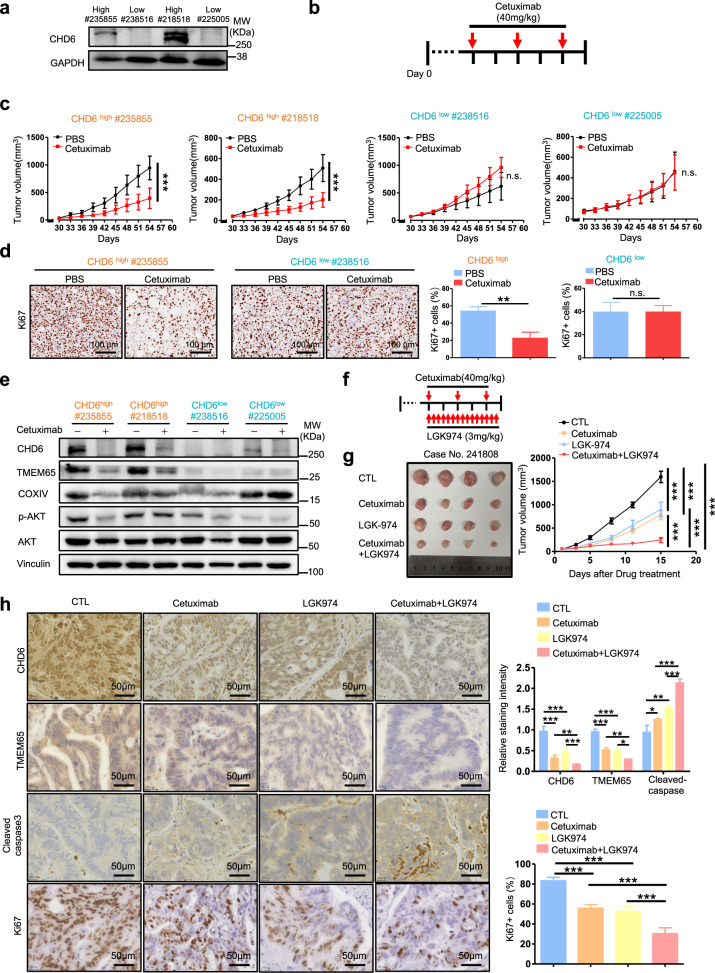
Targeting CHD6 expression via EGFR and Wnt inhibitor suppresses CRC in PDX model. **a** Immunoblot images showing CHD6 expression in four cases of PDX tumors. **b** Schematic diagram of the cetuximab treatment after the establishment of PDX tumors in immunocompromised mice. **c** Growth of the indicated PDX tumors that were treated with either Cetuximab or PBS as control. *n* = 4 biological replicates. Data are presented as means ± SD. ***P < 0.001, ns, not significant. *P* values were calculated by two-way ANOVA. **d** Representative images of IHC staining for Ki67 in PDX tumor sections with the indicated treatments (left) and quantification of Ki67-positive areas (right). *n* = 4, data are presented as means ± SD. **P < 0.01, ns, not significant. *P* values were calculated by two-tailed *t*-test. **e** Immunoblot images showing the indicated protein levels analyzed from the PDX tumors that were treated with either Cetuximab or PBS. **f** Schematic diagram of the Cetuximab and LGK974 combination treatment after the establishment of PDX tumors in immunocompromised mice. **g** Representative images (left) of the PDX tumors that were harvested at the end of the experiment. Growth curves showing the proliferation of PDX tumors in each indicated treatment group (right). *n* = 4 biological replicates. Data are presented as means ± SD. ***P < 0.001. *P* values were calculated by two-way ANOVA. **h** Representative images (left) and quantifications (right) of IHC staining for CHD6, TMEM65, cleaved caspase-3, and Ki67 in PDX tumors from the indicated treatment groups. Data are presented as means ± SD. ***P < 0.001, **P < 0.01, *P < 0.05. *P* values were calculated by one-way ANOVA.

To validate the relevance of our findings to human CRC and to further examine whether hindering CHD6-TMEM65 signaling axis can control the capacity of tumor formation in human CRC, we established PDXs^40^ by implanting primary tumor samples resected from CRC patients into the immunocompromised mice. Two CRC PDX sets (all have WT *RAS* gene) were established, and the expression levels of CHD6 were characterized. Two PDXs contain high CHD6, while the other two PDXs contain low CHD6 (Fig. 8a). As EGF signaling positively regulates CHD6 expression, we rationalized that anti-EGFR monoclonal antibody Cetuximab can be exploited for treatment of CHD6-overexpressing CRC (Fig. 8b). Significantly, administration of the Cetuximab in the established CHD6-high PDX tumors can mitigate tumor progression effectively as revealed by the reduced tumor volume and Ki67 staining (Fig. 8c, d). By contrast, Cetuximab had a minimal impact on reducing tumor volume and Ki67 staining of CHD6-low PDX tumors (Fig. 8c, d) even though they all have WT *RAS* gene. The administration of Cetuximab in CHD6-high group dramatically diminished the expression of TMEM65, COXIV while Cetuximab’s impact on CHD6-low group is less effective as demonstrated by immunoblotting (Fig. 8e).

Importantly, these data show that the role of CHD6 in activating TMEM65 pathway and promoting tumorigenesis can be recapitulated in PDX CRC clinical samples. We additionally illustrated that the regulation network of EGF-AKT signaling in stabilizing CHD6, and consequent accumulation of TMEM65 during cancer formation can be blocked by Cetuximab treatment. Cetuximab is frequently used to treat CRC patients with WT Ras/Raf. However, among these patients, only half respond well, suggesting that other gene status needs to be considered. Our PDX experiments demonstrate the effectiveness of Cetuximab in suppressing tumor growth in CHD6-high CRC and also point out that in addition to WT Ras/Raf status, expression level of CHD6 is critical for determining the treatment efficacy of Cetuximab.

Our data that in addition to EGF, CHD6 is also upregulated by Wnt signaling support the exploration of Cetuximab in combination with Wnt inhibitor in treating CRC exhibiting high CHD6 expression. Next, we set up a CRC PDX model for drug combination efficacy assays and found that the combination of Cetuximab and Wnt inhibitor (LGK-974) was more efficient in hindering tumor growth than Cetuximab or the LGK-974 inhibitor alone in high CHD6-expressing PDX (case no: 241808) as revealed by the reduced tumor volume, diminished TMEM65 staining, increased cleaved caspase-3 staining, and decreased Ki67 staining (Fig. 8f–h). Together, Cetuximab as monotherapy and Cetuximab plus LGK-974 inhibitor as combination therapy may be considered for therapeutic design for CHD6-high, Ras/Raf WT CRC patients.

## Discussion

**Fig. 9:**
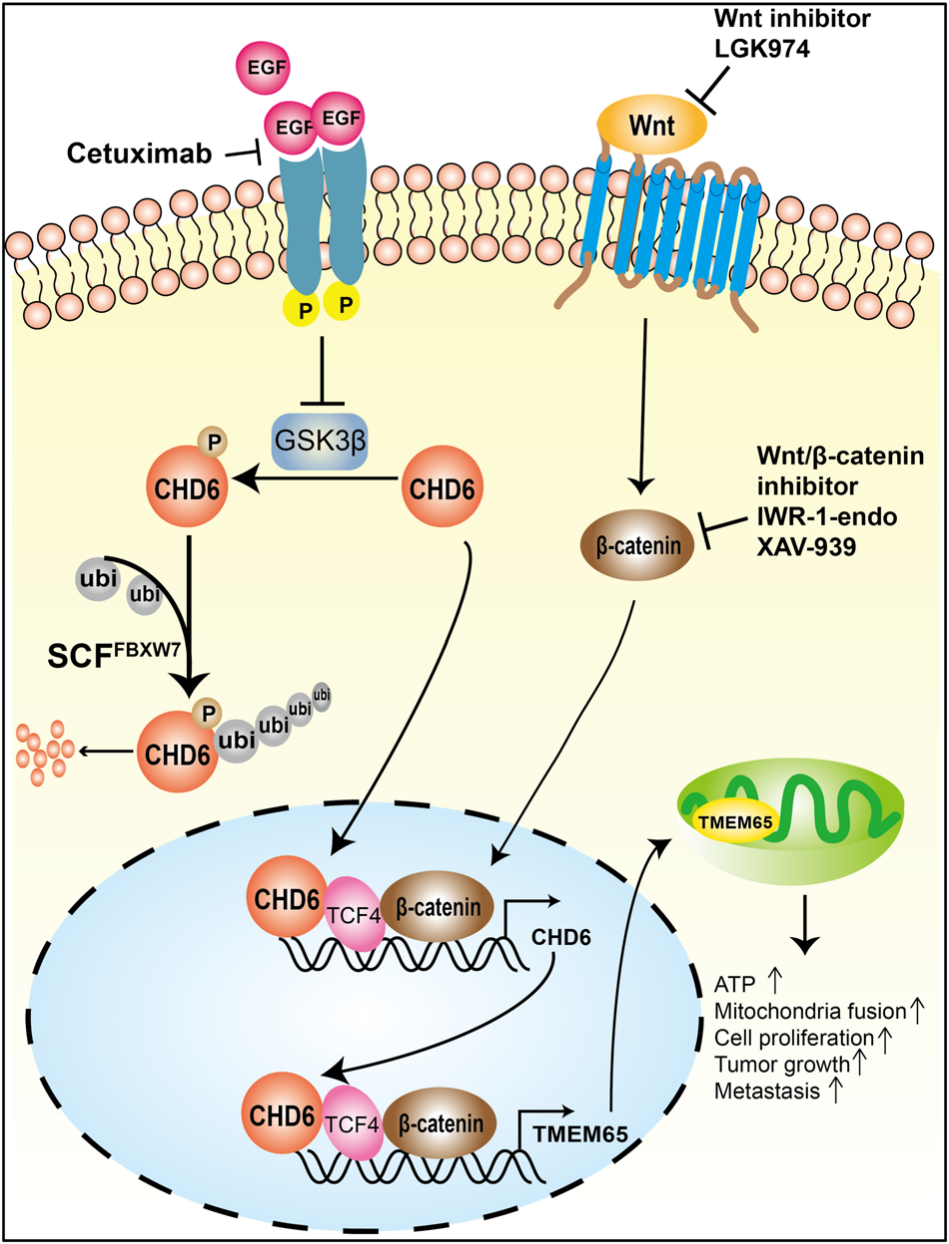
CHD6-TMEM65 axis is regulated by both EGF and Wnt signaling pathways in promoting cancer growth. Model depicts that CHD6 regulates mitochondrial functions by promoting transcription of *TMEM65* in response to EGF and Wnt stimulation. Upon activation of the EGFR-GSK3β axis, CHD6 is not vulnerable to FBXW7-mediated ubiquitination and is thus stabilized. Under the activation of Wnt signaling, β-catenin/TCF4 directly bind to the promoter of *CHD6* to enhance its transcription. CHD6 in turn facilitates transcriptional activity of β-catenin/TCF4 (Wnt signaling) to enhance the expression of *TMEM65*, thereby facilitating tumorigenesis via regulating mitochondrial dynamics, ATP production, and metastasis.

CHD family members, including CHD6, are involved in gene regulation through chromatin remodeling in an ATP-dependent manner. They use chromodomains to bind histone tails and employ the SWI2/SNF2-like ATPase/helicase domain to remodel chromatin by moving histones, but our knowledge about upstream regulators and downstream targets of CHD6 has not been characterized. EGF-PKB/AKT signaling is highly activated in CRC, and the signal targets are not completely known. Here we characterize that CHD6 is overexpressed in CRC and is a critical regulator of Wnt-TCF4 signaling involved in cell proliferation and promoting tumorigenesis. We show that EGF, GSK3β, FBXW7, and Wnt have activities in regulating CHD6 expression. FBXW7 is identified as an E3 ubiquitin ligase that binds and destabilizes CHD6. Notably, CHD6 deregulates mitochondrial homeostasis by positively regulating *TMEM65* gene expression through Wnt and TCF4 signaling. Our data shed light on CHD6 upstream regulatory circuit and reveal how EGF/Wnt oncogenic signal promotes CHD6’s downstream activity toward TMEM65 pathway to cause deregulation of mitochondrial dynamics and subsequently promote cancer metastasis and tumorigenesis (Fig. 9).

### CHD6 is a cancer-associated marker

*CHD6* has been mapped to a region of amplification in CRC^41^. It is mutated in both CRC and bladder cancer^42,43^. However, these defects were not further characterized and biological activities in cancer were not unveiled. Our data fill this knowledge gap by identifying for the first time that CHD6 is highly expressed in CRC, and by characterizing its oncogenic activities including impacts on cell proliferation, migration, invasion, mitochondrial homeostasis, and tumorigenesis. CHD6 interacts with several proteins, such as MLL, CDK19, BRD7, and CTCF, but the significance of these interactions remains largely unknown^44^. It remains to be characterized whether the association of these proteins is critical for CHD6’s oncogenic role. It is important to point out that despite the high degree of sequence identity (~50%) among the group III subfamily members (CHD6–9), so far these members share non-redundant functional roles in the cell. Other than CHD6, roles of other members in CRC development required further investigation. Importantly, as indicated in our analysis (Fig. 1a), *CHD6* is mutated in CRC. Indeed, we identified a cancer-specific missense mutation (P2128L) of *CHD6* on FBXW7 substrate binding site from CRC patients. However, the significance and functional impact of this mutant have not been elucidated. We demonstrated that this cancer-derived *CHD6* mutant is resistant to FBXW7-mediated degradation, suggesting that its continual stability/activity is critical in mediating tumorigenesis.

### Establishing intestine tissue-specific *CHD6*-knockout mice

CHD6 Δexon12 (to delete the ATPase domain) mice have been characterized^45^. The deletion leads to cerebellar defects and ataxia. So far, no tissue-specific *Chd6*-knockout mouse is employed to investigate its role in cancer. CHD6’s impact on human disease has been documented in a chromosomal translocation t (18;20) (q21.1; q11.2) event between *CHD6* (located at 20q12) and the basic helix-loop-helix transcription factor 4 gene (located at chromosome 18), which resulted in mental retardation syndrome^46^. However, CHD6’s biological role remains an enigma. We set up a *Chd6* CKO mice by using the CRISPR-Cas9 genome-editing system to understand the genetic contribution of *Chd6* during the development of tumorigenesis. Villin-specific knockout of *Chd6* using Cre-loxP system led to slower formation of cancer upon the treatment of AOM/DSS, suggesting the critical role of Chd6 in promoting the growth of colon cancer. Our CRISPR-mediated knockout of *Chd6* can be utilized in other types of cancer studies if using proper Cre expressing system, such as liver or prostate cancer. It would be a useful tool to understand the roles of CHD6 in other cancers.

### EGF signaling in decelerating CHD6 degradation

EGF/AKT signaling is highly active in many types of cancers, and it becomes a critical therapeutic target for anti-cancer drugs. Although AKT activation is frequently observed in advanced clinical stages in many types of cancers, the consequence or target of aberrant activation of AKT remains not fully understood. GSK3β is inhibited through AKT-dependent phosphorylation. Our studies demonstrate that GSK3β mediates CHD6 phosphorylation, which destabilizes CHD6 through enhancing FBXW7-mediated CHD6 ubiquitination. Therefore, EGF/AKT activation can increase the stability of CHD6 to drive the activity of downstream signals. The fact that FBXW7 mediates CHD6 degradation is reminiscent of the regulatory mechanism of many FBXW7 targets. For examples, FBXW7 binds to SREBP phosphorylated by GSK3β. The binding leads to SREBP degradation^47^. FBXW7 is a tumor suppressor^48^ protein through recognizing and degrading several oncoproteins, including FOXO4, cyclin E, c-JUN, Aurora B, and MYC^20,24,31,32^. It is reasonable to have a tumor suppressor E3 ligase FBXW7, which is frequently mutated in colon cancer, to degrade a potential oncogenic protein CHD6. Our study adds a new member to the target list of FBXW7 E3 ligase.

### Role of CHD6-TMEM65 axis in regulating mitochondrial dynamics

TMEM65 is localized at the mitochondrial inner membrane^49,50^, but little is known about its regulation or other biological functions. Noticeably, TMEM65 is overexpressed in cancer, which is consistent with the role of its upstream regulator CHD6 as CHD6 is also highly expressed in CRC.

Mitochondria are very dynamic organelles. They constantly undergo fission and fusion to impact the shape, size, number of mitochondria, subcellular transport, mitochondrial quality control (mitophagy), and programmed cell death (apoptosis) in the cell. The balance of fission and fusion events manifested mitochondrial dynamics, which is regulated by a number of mitochondria-shaping proteins such as Drp1. Drp1 (DNM1L) has a role in mitochondrial fission via mediating membrane fission through its oligomerization and interacting with membrane-associated tubular structures. We showed that TMEM65 can downregulate p-Drp1, suggesting that TMEM65 may have a role in antagonizing mitochondrial fission. Activated fission and/or inhibited fusion results in fragmented mitochondria, whereas active fusion and/or inhibited fission leads to mitochondrial elongation. Indeed, *CHD6* KD leads to upregulation of p-Drp1, which in turn causes fragmented mitochondria (fission), while TMEM65 expression can reverse this impact caused by *CHD6* KD. That is, TMEM65 can downregulate p-Drp1 to manifest mitochondrial elongation (fusion). Determining how CHD6/TMEM65 can downregulate p-Drp1 warrants further investigation. Fused mitochondria seem to promote increased oxidative metabolism, ATP production, and reduced ROS. These observations mediated by CHD6/TMEM65 expression are consistent with the role of regulated mitochondrial dynamics in cancer metastasis and tumorigenesis^51,52^. It is obvious that CHD6/TMEM65 expression has a role of increasing ATP production in mitochondria (bioenergetics) and another role of resisting apoptosis. It is then important to point out that both aspects are regulated by mitochondria^53^ and are critical in cancer development.

In addition, mitochondrial deregulation results in a variety of diseases^54^. It is conceivable that CHD6/TMEM65 deregulation may have a pathological impact on these diseases. For example, defects in TMEM65 lead to a mitochondrial disorder manifested as a complex encephalomyopathic phenotype. Further, TMEM65 interacts with connexin 43 (Cx43)^55^, and its KD affects the protein level of Cx43^55^ to regulate heart function. It remains to be determined whether CHD6 affects the expression of Cx43.

We observed that TMEM65 overexpression leads to more cristae in mitochondria compared to controls. This increase of cristae number could result in higher levels of respiratory chain proteins and the ATP synthase^56^, thereby boosting oxidative phosphorylation activity. This phenomenon is consistent with the role of CHD6/TMEM65 in increasing OCR and ATP production.

### CHD6 interacts with TCF4 to regulate Wnt signaling

CHD6 interacts with TCF4 and β-catenin, implying its role in Wnt signaling. Its interaction with TCF4 is reminiscent of the observation that CHD6 functions as an epigenetic modulator, coordinating chromatin structure for CFTR expression^44^. We showed that CHD6 engages in the Wnt signaling pathway and modulates *TMEM65* expression through its binding to *TMEM65* promoter in a TCF4-collaborating manner. Interestingly, *CHD6* is positively regulated by Wnt signaling as its expression is upregulated by TCF4 activity and Wnt ligand. Given that EGF can also stabilize CHD6, it is then conceivable that CHD6 participates in both EGF and Wnt signaling pathways, which have major impacts in promoting the development of CRC (Fig. 9).

### Cetuximab treatment in CHD6-high CRC

Cetuximab is frequently used to treat CRC patients based on the WT *Ras* gene status. Nevertheless, among these patients, only half can respond well, suggesting that more detailed studies are required to understand the discrepancy. We found that Cetuximab has different treatment efficacies in suppressing tumor growth based on the expression level of CHD6 in two sets of WT Ras PDX models. Treating CHD6-high PDX tumors with Cetuximab can mitigate tumor progression effectively. In contrast, Cetuximab had a less impact on the growth of CHD6-low PDX tumors even though these tumors have WT *Ras* gene status. Our results may help explain why not all WT Ras CRC tumors respond to Cetuximab well. We interpret these results as an indication that the status of CHD6 needs to be considered before the administration of Cetuximab. Given that both EGF and Wnt signaling pathways can positively regulate CHD6 activity, it lends credence to the possibility that targeting EGFR-ERK activation (Cetuximab) plus Wnt signaling (Wnt inhibitor) might have a better synergistic effect in treating CHD6-high CRC. Indeed, Cetuximab plus Wnt inhibitor LGK-974 as a combination treatment strategy for CHD6-high, Ras/Raf WT CRC PDX studies demonstrated the feasibility (Fig. 8f–h). Our findings bear important prognostic and therapeutic implications for the treatment of CRC.

Taken together, our data uncover a link of EGF signaling, GSK3β/FBXW7 regulation, CHD6 stability, TMEM65 expression, mitochondrial dynamics, Wnt signaling, and tumorigenicity. The role of EGF/GSK3β in regulating CHD6 stability through FBXW7 and the activity of Wnt/TCF4/β-catenin signaling in regulating *CHD6* transcription highlight important layers of regulation on the activation of CHD6 during tumorigenicity. Further development of compounds that promote FBXW7-mediated CHD6 degradation and inhibit Wnt signaling can be a rational cancer therapy for CHD6-overexpressing cancers.

## Materials and methods

### Animal studies

All animal experimental procedures were approved by the Animal Ethical and Welfare Committee of Sun Yat-sen University.

#### Mice and genotyping

*Chd6*^flox/flox^ mice were established via CRISPR/Cas9 system. *Chd6*^flox/flox^ mice were intercrossed with *Villin*-*Cre*^ERT^ mice to obtain *Chd6*^flox/flox^;*Villin-Cre*^ERT^ mice. All mouse lines were maintained on a C57Bl/6 genetic background. For genotyping, tail or colon tissues were cut into 2-mm pieces and digested with 50 μL tail buffer at 95 °C for 5 min, followed by incubation at room temperature until the samples were completely digested. Samples were then incubated with 1 μL proteinase K at 55 °C overnight, followed by heat-inactivation at 95 °C for 5 min. Ethanol was used to wash and precipitate genome DNA. 1 μL of purified genomic DNA was used in the PCR genotyping reaction. The amplified products were detected by agarose gel electrophoresis.

#### AOM/DSS model

Two groups of mice were confirmed by genotyping: *Chd6*^flox/flox^ (6 mice) and *Chd6*^flox/flox^;*Villin*-*Cre*^ERT^ (6 mice). For tumor induction, 6-week-old mice were injected with 10 mg/kg AOM (Sigma) intraperitoneally (i.p.) one time at the beginning of the experiment (day 0). On day 5, 2% DSS (MP Biologicals) was given in drinking water for 7 days followed by regular drinking water for 2 weeks. This cycle (7 days DSS plus 14 days water) was repeated twice with 1.5% DSS. Mice were sacrificed at 80 days after AOM injection. To induce *Chd6* knockout, mice were administered with 50 mg/kg tamoxifen (Sigma, 10 mg/mL in 90% corn oil) for 5 days 2 weeks before AOM injection.

#### Xenograft model

Xenograft cancer models were established as previously described^57,58^. Briefly, 5-week-old female BALB/c nude mice were purchased from GemPharmatech Co., Ltd. 1 × 10^6^ HCT116 cells, containing stably tetracycline-inducible sh*CHD6* construct, were subcutaneously injected into the right flank. When tumor volumes reached 50–80 mm^3^, all the tumor-bearing mice were randomly divided into two groups of 6 animals per group. Animals were either i.p. injected with doxycycline (50 mg/kg, Selleck) or vehicle every three days. Tumor widths and lengths were measured twice per week using a caliper. Tumor volumes were calculated by the formula: V (mm^3^) = (width^2^ × length)/2.

#### Liver metastasis model

1 × 10^6^ HCT116 cells, containing stably tetracycline-inducible sh*CHD6* with or without TMEM65 overexpression, were injected into the spleen of BALB/c nude mice under general anesthesia. The mice in *CHD6* KD group were i.p. injected with 50 mg/kg doxycycline every three days. On day 30, mice were sacrificed by CO_2_ inhalation.

#### The PDX model

PDX experiments were performed as previously described^59^. Basically, four KRAS/BRAF WT PDX lines were selected. Patient-derived tumor tissues were cut into small fragments (3 mm^3^) and subcutaneously implanted into immunocompromised mice (GemPharmatech Co., Ltd.). For single drug treatment, when tumor volume reached ~50 mm^3^, mice were randomly allocated to two groups and were i.p. injected with either cetuximab (40 mg/kg, Selleck) or vehicle once a week. For combination treatment, when tumor volume reached ~50 mm^3^, mice were randomly allocated to the following treatment groups: (1) vehicle control; (2) cetuximab (40 mg/kg, Selleck); (3) LGK974 (3 mg/kg, Apexbio), (4) cetuximab and LGK974 using doses described above. Administration of cetuximab was the same as addressed above. LGK974 was administered to mice via i.p. injection every day. Mouse weights and tumor volumes were measured every three days during the experiments.

### TEM

HCT116 cells and mouse colon tissues were fixed with fixation solution (Servicebio, G1102). After 24 h, samples were washed 3 times with 0.1 M cacodylate buffer and post-fixed with 1% osmium tetroxide (Ted Pella Inc) for 2 h at room temperature. Then the samples were dehydrated in a graded ethanol series and embedded into SPI-Pon 812 (Structure Probe, Inc.). The embedding models were polymerized at 60 °C for 2 days. Ultrathin sections (60–80 nm) were cut with a Leica Ultracut microtome (Leica UC7) and stained with 2% uranyl acetate and 2.6% lead citrate. TEM imaging was performed on a Hitachi TEM system (HT7800). The lengths of mitochondria in TEM images were measured using ImageJ.

### Organoid culture

Human CRC organoid was cultured as described previously^60,61^. In brief, fresh CRC tumor tissues were washed with cold PBS containing penicillin-streptomycin, and cut into 3–5 mm fragments. Pieces were digested with EDTA (5 mM) on ice for 60 min with mixing. After being digested into clumps of cells, the sample was mixed with Matrigel and seeded into a 24-well plate. After Matrigel polymerization (10 min at 37 °C), 500 μL/well advanced DMEM/F12 medium containing 10 mM HEPES, 100 U/mL penicillin/streptomycin, 2 mM GlutaMAX, 1× B27, 1× N2 (Life Technologies), 10 nM gastrin I (Biogems), 500 ng/mL R-spondin1 (Peprotech), 10 μM SB202190 (Sigma), 10 μM Y-27632 (Abmole), 50 ng/mL recombinant EGF, 500 nM A83-01 (Biogems), 100 ng/mL recombinant Noggin (Peprotech), 10 mM nicotinamide (Sigma), 1 mM N-acetylcysteine (Sigma) was added to each well containing organoids. On the second day, the organoids were transduced with shCTL or sh*CHD6* lentivirus using polybrene (10 μg/mL) (Millipore, TR-1003-G).

### Cell culture and transfection

All colorectal cell lines and HEK293T cells used in this study were obtained from ATCC, confirmed to be mycoplasma-free, and incubated in humidified incubator at 37 °C with 5% (vol/vol) CO_2_. HCT116, SW620, and HEK293T cells were cultured in Dulbecco’s modified Eagle’s medium (DMEM) supplemented with 10% FBS. DLD-1 cells were cultured in RPMI 1640 medium (RPMI) with 10% FBS. Lipofectamine^TM^ 2000 (Invitrogen) and polyethylenimine (Polysciences, 24765) were used for cell transfection in this study following the manufacturer’s standard protocol.

### Plasmids, cloning, and lentivirus production

The cDNAs for *CHD6*, *TMEM65*, *TCF4,* and *FBXW7* were amplified by PCR. These cDNAs were cloned into a pCMV5 vector to generate fusion proteins with N-terminal Flag or Myc tag. *CHD6* cDNA was also subcloned into pEGFP-N1 vector (Addgene) to produce a fusion protein with a GFP tag at the C-terminus of CHD6. TMEM65-Flag was also re-cloned into a lentiviral vector pWPI. CHD6 mutant was generated by using a Fast Mutagenesis Kit V2 (Vazyme) according to the manufacturer’s instructions. The resulting plasmids were verified by sequencing.

For *CHD6* KD, lentiviral shRNA constructs were purchased from (GeneCopoeia). We screened 6 hairpins targeting human *CHD6* transcripts and found two independent sequences that reduced protein levels by > 80% (*CHD6* shRNA #31: 5′-GCACAGAAGATCAAGCGATTT-3′; *CHD6* shRNA #32: 5′-GCGAGTATAAGAACAGTAACC-3′). For the doxycycline-inducible *CHD6* KD, the shRNAs targeting *CHD6* were inserted into Tet-pLKO-puro vector (Addgene). Tet-pLKO-sh*CHD6*-1 (*CHD6* shRNA #1: 5′-CATTCCAGCAATCATAGTTAA-3′) was designed to target 3′UTR of *CHD6*. The shRNA sequence in Tet-pLKO-sh*CHD6*-2 is the same as sh*CHD6*-31 (5′-GCACAGAAGATCAAGCGATTT-3′). For the doxycycline-inducible FBXW7 KD, the shRNA sequence (5′-TGATACATCAATCCGTGTTTG-3′) was inserted into Tet-pLKO-puro vector. For *TMEM65* KD, the sequence (5′-TCCAGGTTAGGCCTGTCAATT-3′) was inserted into pLKO.1 puro vector.

To prepare lentivirus, HEK293T cells were seeded into a 10 cm dish at a density of 1 × 10^7^, and were co-transfected with 10 μg shRNA lentiviral plasmid, 5 μg psPAX2 and 5 μg pMD2.G using polyethylenimine (Polysciences, 24765). The supernatant containing lentivirus was collected at 48 and 72 h after transfection and were filtered through Millex-GP Filter Unit (0.45 μm pore size, Millipore). HCT116, DLD-1, and SW620 cells were infected twice with filtered viral supernatant containing 10 μg/mL polybrene (Millipore, TR-1003-G).

### Western blot analysis

Cells were washed twice with cold PBS and lysed in RIPA buffer (50 mM Tris-HCl, pH 7.5, 150 mM NaCl, 0.5 mM EDTA, 0.1% SDS, 1% Triton X-100, 1% sodium deoxycholate, phosphatase and protease inhibitors). Sample proteins were separated by SDS-PAGE and transferred onto PVDF membranes. For protein turnover assay, cells were treated with 60 μg/mL cycloheximide (MDBio, Inc.) for the indicated times before collection. The following antibodies were used in the immunoblotting and immunoprecipitation experiments: CHD6 (1:500, Abcam, ab114095), CHD6 (1:1000, Santa Cruz, sc-393445), TMEM65 (1:500, Sigma, HPA025020), p-AKT (Ser473) (1:1000, Cell Signaling, 4060S), AKT (1:2000, Cell Signaling, 2920S), Vinculin (1:4000, Cell Signaling, 4650S), FBXW7 (1:5000, Abcam, ab109617), COX IV (1:5000, Cell Signaling, 4850), PPOX (1:2000, Santa Cruz, sc-271768), VDAC1 (1:2000, Santa Cruz, sc-390996), mtTFA (1:1000, Santa Cruz, sc-166965), p-Drp1 (Ser616) (1:800, Cell Signaling, 4494S), Drp1 (1:2000, Proteintech, 12957-1-AP), Parkin (1:1000, Proteintech, 14060-1-AP), GAPDH (1:4000, Proteintech, 60004-1-Ig), Flag-tag (1:5000, Sigma, F1804), HA-tag (1:5000, Cell Signaling, 3724S), Myc-tag (1:5000, Cell Signaling, 2276S), β-Catenin (1:4000, BD, 610153), TCF4 (1:1000, Santa Cruz, sc-166699), GSK3β (1:4000, Cell Signaling, 9832S).

### Immunoprecipitation

Cells were lysed in IP lysis buffer (50 mM Tris-HCl, pH 7.5, 150 mM NaCl, 0.1% Triton X-100, 0.1% NP-40, 1 mM EDTA, phosphatase and protease inhibitors). Lysates were incubated either with CHD6 antibody or IgG at 4 °C overnight. Protein A/G agarose beads (Santa Cruz, sc-2003) were added into each sample and then incubated at 4 °C for 3 h. Samples were washed 4 times using cold IP lysis buffer and boiled with 2× SDS loading buffer. For Flag-tag immunoprecipitation, cell lysate supernatants were incubated with Flag-M_2_ agarose beads at 4 °C overnight.

### Immunofluorescence

Frozen sections were blocked in 2% bovine serum albumin for 1 h at room temperature. After blocking, sections were incubated with CHD6 (1:10, Santa Cruz, sc-393445) and TMEM65 (1:100, Sigma, HPA025020) antibodies overnight at 4 °C. The samples were subsequently incubated with Alexa Fluor 488- and Alexa Fluor 594-conjugated secondary antibodies. Nuclei were stained with DAPI (Invitrogen). Fluorescence signals were imaged using fluorescence microscope (Leica).

### Ubiquitination assay

For CHD6 ubiquitination assay, cells were transfected with the indicated plasmids for 48 h. MG132 (10 μmol/mL, Selleck) was added to the culture media for 6 h. Then cells were collected and lysed in Buffer A (6 M guanidine-HCl, pH 8.0, 10 mM imidazole, 0.1 M Na_2_HPO_4_/NaH_2_PO_4_). Cell lysates were incubated with Ni-NTA agarose (Invitrogen) at room temperature for 4 h and washed with Wash Buffer (25 mM Tris-HCl, pH 6.0, 20 mM imidazole). Eluted proteins were analyzed by SDS-PAGE and immunoblotting with the indicated antibodies.

### Histopathological scoring

Histopathological conditions were scored in a blinded fashion using a method previously published with minor modifications^62^. Briefly, the condition of ulcerative colitis and dysplasia of each mouse colon were graded ranging from scales 0 to 5 according to the following criteria: 0, no changes; 1, minimal inflammatory cell infiltration, with or without minimal epithelial dysplasia; 2, mild to diffuse inflammatory cell infiltration with occasional spreading to the submucosa with erosions, minimal to mild mucin depletion and epithelial dysplasia; 3, mild to moderate inflammatory cell infiltration that were sometimes transmural with ulceration, moderate dysplasia, and mucin depletion; 4, marked inflammatory cell infiltration that were often transmural and associated with ulceration, marked dysplasia and mucin depletion; 5, marked transmural inflammation with severe ulceration, gland loss and dysplasia.

### Immunohistochemistry

Immunohistochemistry was performed following a standard protocol. Paraffin-embedded sections were dewaxed in xylene and rehydrated in gradient ethanol. Antigen retrieval was performed using 0.01 M sodium citrate in a high-pressure cooker. After cooling down, sections were incubated in 3% H_2_O_2_ for 10 min and blocked with goat serum for 1 h, followed by primary antibody incubation at 4 °C overnight. On the second day, the sections were incubated with secondary antibodies for 15 min at room temperature and developed with diaminobenzidine. Ki67 (Cell Signaling, 9449), CHD6 (Santa Cruz, sc-393445), TMEM65 (Sigma, HPA025020), COX IV (Cell Signaling, 4850), PPOX (Santa Cruz, sc-271768), p-Drp1(Ser616) (Cell Signaling, 4494S), OPA1 (Santa Cruz, sc-393296) and cleaved Caspase-3 (Asp175) (Cell Signaling, 9664) antibodies were used.

### RNA isolation and RT-qPCR

Total RNA was extracted using TRIzol Reagent (Invitrogen, #15596026) according to the manufacturer’s protocol, and reverse-transcribed into cDNA using ReverTra Ace® qPCR RT Master Mix (TOYOBO). The expression of target genes was detected using the 2× SYBR Green qPCR Master Mix (Biotool, #B21203) by a LightCycler® 480 II (Roche).

### OCR

Prior to assay, cells were seeded on XF24 microplate (5 × 10^4^ cells per well) and cultured at 37 °C and 5% CO_2_ overnight. Sensor cartridge was hydrated in calibrant and incubated in a 37 °C, non-CO_2_ incubator. For the XF Cell Mito Stress Test Kit (Agilent, 103015-100), assay medium was prepared by supplementing XF Base Medium (Agilent, 102353-100) with 10 mM glucose (Sigma, G7528), 1 mM pyruvate, and 2 mM glutamine. Cells were washed with assay medium twice, and incubated at 37 °C, non-CO_2_ incubator for 45 min. Oligomycin, FCCP and Rotenone/antimycin A were each resuspended in assay medium and added to the microplate. The OCR was determined by a Seahorse XF24 analyzer (Agilent Technologies Co., Ltd.).

### ATP assay

#### Total ATP production

The total ATP was detected using ATP Bioluminescence Assay Kit CLS II (Roche, #11699695001). 100 μL of cell suspension (1 × 10^6^ cells/mL) was diluted to 9 volumes of boiling TE buffer (100 mM Tris, 4 mM EDTA, pH 7.75) and was incubated at 100 °C for 2 min. After incubation, samples were centrifuged at 1000× *g* for 1 min, and 50 μL supernatant of each sample was transferred to a well of 96-well microplate. ATP level was detected by adding 50 μL luciferase to each sample, and the bioluminescent signal was measured with a Microplate Reader (BioTek Instruments, Inc.).

#### Glycolytic and mitochondrial ATP production

For the determination of glycolytic ATP production rate (glycoATP Production Rate) and mitochondrial ATP production rate (mitoATP Production Rate), Agilent Seahorse XF Real-Time ATP Rate Assay Kit (103592-100) was used according to the manufacturer’s protocol. The glycoATP production rate is equivalent to Glycolytic Proton Efflux Rate (Glucose + 2 ADP + 2 Pi = 2 Lactate + 2 ATP + 2 H_2_O + 2 H^+^). ECAR data indicates the total proton Efflux Rate, and the mitochondrial Proton Efflux Rate can be calculated according to the ECAR that is changed by adding rotenone and antimycin A. The mitoATP Production Rate is calculated according to Equation: mitoATP Production Rate (pmol ATP/min) = OCR^ATP^ (pmol O_2_/min) × 2 (pmol O/pmol O_2_) × P/O (pmol ATP/pmol O). OCR^ATP^ can be calculated as the OCR that is inhibited by adding oligomycin (ATP synthase inhibitor). Assay medium was prepared by supplementing XF Medium-phenol free, pH 7.4 (Agilent, 103575-100) with 10 mM glucose (Sigma, G7528), 1 mM pyruvate (Gibco®, 11360070), 2 mM glutamine (Gibco®, 25030081). Oligomycin, Rotenone, and antimycin A were prepared prior to assay.

### Heme content (PPIX fluorescence)

Heme content measurement assay was performed as described previously^63^. Briefly, cells were collected and resuspended in 500 μL of 20 mM oxalic acid (Sangon, A610400) and stored in a closed box at 4 °C overnight. On the second day, 500 μL of 2 M oxalic acid (heat for dissolution) was added and mixed with the stored samples. 500 μL of the mixture was heated at 98 °C for 30 min, and the remaining samples were kept at room temperature. All prepared mixtures were centrifuged at 16,000× *g* for 2 min. 200 μL of mixture from each sample was transferred to a black microtiter plate and the fluorescence (Excitation: 400 nm; Emission: 620 nm) was measured in a fluorescence microplate reader (BioTek Instruments, Inc.). The fluorescence of corresponding unboiled samples was used to correct for background fluorescence.

### L-Wnt3a CM

L-Wnt3a-expressing cells were seeded into 10 cm dish, and cultured in DMEM supplemented with 10% FBS. After 4 days, cell culture supernatant was collected, filtered, and kept at 4 °C. The cells were cultured for another 3 days for a second supernatant collection. The second supernatant was combined with the first collection and stored at −80 °C.

### ChIP-qPCR

ChIP was performed as previously described^58,64^. Briefly, control and *CHD6* KD HCT116 cells were cross-linked using 1% formaldehyde for 20 min at room temperature. Reactions were quenched by treating with 0.125 M glycine for 5 min at room temperature. Then cells were washed with cold PBS and collected in 1 mL ChIP lysis buffer (5 mM HEPES, pH 8.0, 85 mM KCl, 0.5% NP-40, protease inhibitors). Chromatin fragmentation was performed using a Diagenode BioruptorPico sonicator (30 s on, 30 s off for 10 cycles). Clear chromatin extracts were incubated with 20 μL Magna ChIP™ Protein A + G Magnetic Beads (Millipore, 16–663) and 2 µg antibody (CHD6, Santa Cruz, sc-393445; β-Catenin, BD, 610153; TCF4, Santa Cruz, sc-166699) or IgG overnight at 4 °C. Immunoprecipitated chromatin was washed with low-salt buffer (0.1% SDS, 1% Triton X-100, 2 mM EDTA, 20 mM Tris-HCl, pH 8.1, 150 mM NaCl) twice, washed with high-salt buffer (0.1% SDS, 1% Triton X-100, 2 mM EDTA, 20 mM Tris-HCl, pH 8.1, 500 mM NaCl) twice, and then washed twice with LiCl buffer (10 mM Tris-HCl, pH 8.0, 250 mM LiCl, 1% NP-40, 1% deoxycholic acid and 1 mM EDTA) and once with TE buffer (pH 8.0). Sample beads were resuspended in 50 μL elution buffer (10 mM Tris-Cl, pH 8.0, 5 mM EDTA, 300 mM NaCl, 0.5% SDS) with 5 μL proteinase K (20 mg/mL) at 65 °C overnight. DNA was purified using PCR purification Kit (Omega, D2500-02). Eluted DNA was used for qPCR.

### Cell lysate fractionation analysis

Fractionation of cell lysates by size-exclusion chromatography was performed as previously described with minor modification^58^. Briefly, CHD6-transfected HEK293T cells were lysed in lysis buffer (50 mM Tris, pH 7.5, 150 mM NaCl, 100 mM EDTA, 0.5% NP-40, 0.1% Triton X-100). The cleared supernatants of each cell lysates were run through a size-exclusion column Superose 6 (GE), and proteins were eluted by PBS (0.01 M phosphate, 0.15 M NaCl, pH 7.4) in GE AKTA avant150 chromatography system at a flow rate of 0.4 mL/min. A total of 40 fractions (0.3 mL/fraction) were collected. Collected protein fractions were resolved with SDS-PAGE, followed by immunoblotting with the indicated antibodies.

### Bioinformatics analysis

RNA was extracted from HCT116 cells with or without *CHD6* KD (dox-inducible *CHD6* KD). HG-U133 Plus 2.0 GeneChips (Affymetrix) was used to examine the gene expression profiles according to the manufacturer’s instructions. Analysis of microarray data was performed in R programming environment (http://cran.r-project.org), and the Bioconductor (http://bioconductor.org) R packages simpleaffy (http://bioinformatics.picr.man.ac.uk/simpleaffy) and EnhancedVolcano (https://github.com/kevinblighe/EnhancedVolcano) were used for analyzing Affymetrix data and generating volcano plot. TCGA expression and mutation data were downloaded from cBioportal^65,66^. The CRC datasets (GSE31595, GSE2109, among others) were downloaded from the Gene Expression Omnibus (GEO), the Oncomine, The Cancer Genome Atlas and The Human Protein Atlas databases. For the CRC study, tumors were stratified with median CHD6 expression. The datasets of GSE31595 and GSE2109 were annotated by GSEA (Broad Institute) on KEGG and Cancer Hallmark Pathways databases. The GEO2R web application (https://www.ncbi.nlm.nih.gov/geo/geo2r/) was used to computerize the differentially expressed genes (adjusted *P* value < 0.01) by comparing the gene expression profiles of CHD6-high and -low CRC (GES2109 and GES14333). The resulting gene list was submitted for pathway analysis using the ‘Core Analysis’ function in the IPA (Qiagen Silicon Valley).

### Patient tissue samples and TMA analysis

All cancer patient samples were collected with the patients’ written informed consent and approval from the Institutional Review Board of the Sixth Affiliated Hospital of Sun Yat-sen University. For CHD6 expression level analysis, 18 paired CRC and adjacent normal colon tissues were collected from the Department of Surgery at the Sixth Affiliated Hospital of Sun Yat-sen University. Total RNA was isolated, and *CHD6* mRNA level was detected by RT-qPCR. The human TMAs were purchased from Outdo Biotech (Shanghai Outdo Biotech Co., Ltd.). The TMAs contained 180 human colon tissue cores, including 76 paired specimens of CRC and corresponding normal tissues and 28 identified CRC specimens. The immunostained slides were scanned using Slide Scanning System SQS-1000 (TEKSQRAY). TMA images were analyzed with HALO image analysis software (Indica Labs).

### Statistical analysis

The differences between two groups were measured by Student’s *t*-test (GraphPad Prism 6). Multiple group comparisons were performed by one-way ANOVA followed by Tukey test (comparing all pairs of columns) or Dunnett test (comparing all columns vs control column). The statistical significances about cell proliferation and tumor growth in different groups were calculated by two-way ANOVA. Survival analysis was performed using SPSS 25 by Kaplan–Meier survival curve and verified by log-rank test. The Pearson correlation was calculated using a package written in the R language (http://cran.r-project.org). χ^2^ test was used to examine the relationship between groups. Cox proportional hazards regression for univariate and multivariate analyses were employed to evaluate which clinicopathologic factors had prognostic values. The *P* values and hazard ratios are shown and *P* < 0.05 was defined as statistically significant.

## Supplementary information

Supplementary information

## Data Availability

The array data has been deposited in the GEO database with accession code GSE163124.
