# Supplementary material for: The chromatin remodeler CHD6 promotes colorectal cancer development by regulating TMEM65-mediated mitochondrial dynamics via EGF and Wnt signaling
Source: Cell Discov. 2022 Dec 6;8:130. doi: 10.1038/s41421-022-00478-z (PMC9727023; doi:10.1038/s41421-022-00478-z)
Supplement: Supplementary file 1 — Supplementary information [file 41421_2022_478_MOESM1_ESM.pdf]

The chromatin remodeler CHD6 promotes colorectal cancer development by regulating TMEM65-mediated mitochondrial dynamics via EGF and Wnt signaling

Supplemental Experimental Procedures

Cell proliferation, colony formation, migration, invasion and sphere-formation assay

Cell proliferation assay. Cell proliferation assay was performed using Cell number CountingKit-8 (APExBIO) according to the manufactory's instruction. Briefly, DLD-1, HCT116 and SW620 cells were seeded in 96-well plates at a density of $3×10^3$. At the indicated time points, 10 μl of CCK8 was added to each well and then incubated for 3 hours. Cell proliferation was measured by OD 450nm using microplate reader (BioTek Instruments, Inc.).

Colony formation. 200 cells were seeded in each well of 6-well plates. After 14 days, cells were fixed with 100% methanol for 10 minutes and stained with 0.5% crystal violet in 20% methanol for 30 minutes.

Migration assay. $8×10^4$ cells suspended in 150μl serum-free medium were seeded into 24-well transwell chambers (Corning, NY) and complete medium was added into the lower receiver wells. After 16h, cells were fixed with 4% formaldehyde for 5min and stained with 0.5% crystal violet for 20 minutes. Swabbed the inside of each chamber and counted under microscope. For invasion assay, coated the inside of chamber with Matrigel (BD) before seeding cells. 24 hours later cells were fixed and stained as above.

Sphere-formation assay. $6×10^3$ cells suspended in sphere culture medium (serum-free DMEM/F12 medium supplemented with B27, 20ng/ml basic fibroblast growth factor, and 20ng/ml epidermal growth factor (EGF)) were seeded into $25cm^2$ non coated dishes. After 14 days, sphere numbers in each well were calculated.

Cell cycle assay. Cells were stained with 0.05mg/ml propidium iodide (0.1% sodium

citrate, 0.03% NP-40, 0.02mg/ml RNase A) at 4°C overnight. Fluorescence was measured by flow cytometry (Beckman Instruments, Inc.).

Apoptosis analysis. Cells were suspended in binding buffer containing annexin V-FITC and propidium iodide at 37 °C for 15 minutes. Unstained cells were used as a negative control; cells only stained with annexin V-FITC or PI were used to adjust compensation. After washing with PBS, samples were analyzed by Flow Cytometry (Beckman Instruments, Inc.). FlowJo software (TreeStar Inc. Ashland) was used to analyze flow cytometry data.

Mitochondria analysis

Cells were washed with FBS-free DMEM and incubated with 50nM Mitotracker™ Red CMXRos (Invitrogen, M7512) at 37 °C for 30 minutes. After incubation, cells were washed with PBS twice, fixed with 4% PFA for 15 min, and stained with DAPI for 10 min. The mitochondrial images were acquired using confocal microscope (Leica SP8). Mitochondrial length was analyzed using Image J with MiNA-Mitochondrial Network Analysis plugin. For Mito-Tracker fluorescence intensity assay, cells were collected after Mito-Tracker staining and analyzed with a Flow Cytometer (Beckman Instruments, Inc.).

Mitochondria isolation

Mitochondria was isolated using Cell Mitochondria Isolation Kit (Beyotime, C3601), following the manufacturer's protocol. Mitochondrial proteins were used for immunoblotting.

Luciferase Reporter Assay

For the TMEM65-reporter construction, the TCF4 binding sequence on TMEM65 promoter was cloned into pGL3-Basic-IRES vector (Addgene). 293T cells were co-transfected with TMEM65-reporter and a pGL3 reporter containing Renilla luciferase gene. Firefly and Renilla luciferase activities were measured using the dual-luciferase reporter assay system (Promega, USA) on a Luciferase Activity Reader (BioTek Instruments, Inc.). The TMEM65-reporter luciferase activity was normalized by the Renilla luciferase activity.

Size-exclusion column chromatography

TMEM65-transfected HEK293T cells were lysed in lysis buffer (50 mM Tris pH 7.5, 150 mM NaCl, 100 mM EDTA, 0.5% Nonidet P-40, 0.1% Triton X-100). The cleared supernatants of each cell lysates were run through a size-exclusion column Superdex 200 (GE), and proteins were eluted by PBS (0.01M phosphate, 0.15M NaCl, pH 7.4) in GE AKTA avant150 chromatography system. Fractions (0.3 mL/fraction) were collected. Collected protein fractions were resolved with SDS-PAGE, followed by immunoblotting with indicated antibodies.

Supplementary Fig. S1 CHD6 overexpressed in CRC

a, Genomic alterations of *CHD6* in a variety of cancer from the TCGA pan-cancer data.

b, *CHD6* mRNA levels in CRC from TCGA, Gaedcke (GSE20842) and Skrzypczak (GSE20916) Colorectal database. *CHD6* mRNA level was higher in CRC adenocarcinoma. Data are presented as mean ± SEM. $^{**}P < 0.01$ and $^{***}P < 0.001$. p values were calculated by two-tailed t-test.

c, *CHD6* mRNA levels in colorectal cancer and adjacent normal tissue from 18 patients.

d, Kaplan-Meier plot of relapse-free survival time based on the CHD6 expression level in CRC tissues (P values by log-rank test). High *CHD6* level correlated with poor relapse-free survival.

Supplementary Fig. S2 Depletion of *CHD6* decreased CRC cell proliferation, migration, and invasion

a, Representative immunoblots showing the efficiency of shRNA-mediated *CHD6* knockdown (KD) in DLD-1, HCT116 and SW620 cells.

b, Proliferation of DLD-1, HCT116 and SW620 cells, with or without *CHD6* KD.

c, Colony-formation assay of DLD-1, HCT116 and SW620 cells, with or without *CHD6* KD. Quantitation of colony numbers were shown at the bottom.

d, Representative migration images of DLD-1, HCT116 and SW620 cells, with or without *CHD6* KD. Quantitation of migratory cells were shown at the bottom, and Image J was used to quantify migratory events.

e, Representative invasion images of DLD-1, HCT116 and SW620 cells, with or without *CHD6* KD. Quantitation of invasive cells were shown at the bottom, and Image J was used to quantify invasive events.

Assays were performed with three replicates. p values were calculated by two-way ANOVA (**b**) or one-way ANOVA (**c**-**e**). Data are presented as mean ± SD. $^{***}P<0.001$, $^{**}P<0.01$, $^{*}P<0.05$.

Supplementary Fig. S3 *CHD6* KD attenuates the malignant features of CRC cells: sphere formation, PDO proliferation, cell progression, anti-apoptosis, OCR, ATP production, and mitochondria mass

a, Representative images of sphere formation taken from DLD-1, HCT116 and SW620 cells, with or without *CHD6* KD. Quantitation of sphere numbers shown at the bottom (n=3 technical replicates).

b, Representative images of patient-derived organoid cultures, with or without *CHD6* KD (sh*CHD6*). Quantitation of organoid numbers and size (n=3 technical replicates). Data are presented as mean ± SD. *$P < 0.05$. Scar Bar: 25 μm. p value was calculated by two-tailed t-test. *CHD6* KD decreased number and size of CRC patient derived organoids (PDOs).

c, Representative FACS profiles showing cell cycle of HCT116 (Tet-on-sh*CHD6*) cells that were cultured in medium with or without doxycycline (Left). Quantification of cells in G1, S and G2 phase (Right).

d, Representative FACS profiles showing apoptosis of HCT116 (Tet-on-sh*CHD6*) cells that were cultured in medium with or without doxycycline (Left) and quantification of apoptotic cells by Annexin-V staining (Right).

e, Oxygen consumption rate (OCR) of HCT116 cells with or without *CHD6* KD.

f, Quantification of cellular ATP coupled to glycolysis and oxidative phosphorylation in HCT116 cells with or without *CHD6* KD.

g, FACS analysis of Mitotracker Red staining in HCT116 cells, with or without *CHD6* KD. *CHD6* KD decreased mitochondrial mass.

Assays were performed with three replicates. p values were calculated by one-way ANOVA (**a, f**) or two-tailed t-test (**b-d**). Data are presented as mean ± SD. ***$P<0.001$, **$P<0.01$, *$P<0.05$. Dox, with doxycycline induction; no Dox, without doxycycline induction.

Supplementary Fig. S4 The study of CHD6 in pre-clinical animal models

a, Schematic diagram of the xenograft mouse study shown in Fig. 1**d**. Doxycycline (Dox) was administrated by intraperitoneal injection to induce *CHD6* knockdown in HCT116 cells at the indicated days.

b, Tumor weight of subcutaneous tumors derived from HCT116 (Tet-on-sh*CHD6*) cells, with or without doxycycline treatment. N=6 for each group. Data are presented as mean ± SD. *P < 0.05, p value was calculated by two-tailed t-test.

c, Genotyping of the *Chd6*$^{fl/fl}$ and *Chd6*$^{fl/fl}$/*Villin*CreER^{T} (CKO) mice after tamoxifen treatment.

d, Representative images of Alcian Blue/Periodic Acid Schiff (AB/PAS)-stained colon tissue obtained from *Chd6*$^{fl/fl}$ +TAM and *Chd6*$^{fl/fl}$/*Villin*CreER^{T} +TAM (Top). Scale bars, 50 μm. Quantification of goblet cells per crypt in the colons of mice (Bottom) (20 crypts were counted in total from n=3 mice). To induce *Chd6* knock-out, *Chd6*$^{fl/fl}$ and *Chd6*fl$^{/fl}$/*Villin*CreER^{T} mice were administrated with 50mg/kg tamoxifen (Sigma, 10mg/ml in 90%corn oil) for five days. After three weeks, colon samples were used for Alcian Blue/Periodic Acid Schiff staining. Data are presented as mean ± SD. ns, no significance. p value was calculated by two-tailed t-test.

e, Schematic diagram of the AOM/DSS study shown in Figs. 1**h**-**j**, shown again here for clarity.

f, Body weight changes of *Chd6*$^{fl/fl}$ +TAM (n=6) and *Chd6*$^{fl/fl}$/*Villin*CreER^{T}+TAM (n=6) mice in the course of AOM/DSS-treatment. Data are presented as mean ± SD. **P<0.01, p values were calculated by two-way ANOVA.

g, Representative images of Immunofluorescence staining for Chd6 in the colon sections obtained from *Chd6*$^{fl/fl}$+TAM (n=6) and *Chd6*$^{fl/fl}$/*Villin*CreER^{T}+TAM (n=6) mice treated with AOM/DSS. Red, Chd6; Blue, DAPI.

Dox, with doxycycline induction; no Dox, without doxycycline induction; TAM, tamoxifen

Supplementary Fig. S5 CHD6 regulates mitochondrial biogenesis through TMEM65

a, Correlation of *CHD6* and *TMEM65* expression in TCGA colorectal cancer database, p value was calculated by Pearson's correlation coefficient.

b, Survival analysis of CRC patients (GSE17538 and TCGA), stratified using *TMEM65* expression. P values were calculated by log-rank test.

c, RT-qPCR analysis of *TMEM65* expression in *CHD6* overexpression (Top) or *CHD6* KD (Bottom) HCT116 cells.

d, Immunoblot analysis of TMEM65 expression in *CHD6* overexpression (Top) or *CHD6* KD (Bottom) HCT116 cells.

e, Growth curves of HCT116 cells transduced with either vector or *TMEM65* (Top). Immunoblot images confirmed the overexpression of TMEM65 in HCT116 cells (Bottom).

f, Protoporphyrin IX levels in shCTL or sh*CHD6* HCT116 cells. *CHD6* KD cells exhibited decreased level of heme.

g, Protoporphyrin IX levels in HCT116 cells transfected with TMEM65. TMEM65 expression leads to increased heme (Protoporphyrin IX Fluorescence).

h, Immunoblot analysis of PPOX expression in TMEM65 overexpression or knockdown HCT116 cells. TMEM65 expression increased PPOX while TMEM65 KD decreased protoporphyrinogen oxidase (PPOX).

i, 293T cells transfected with Flag-tagged TMEM65 were immunoprecipitated with an anti-Flag antibody, and immunoblotted with indicated antibodies. WCL: whole cell lysates.

j, Representative immunoblot analysis of protein complexes eluted from gel filtration column. Cell lysates were fractionated using Superdex 200 (GE) column. Eluted fractions were collected and revealed by immunoblotting with indicated antibodies. TMEM65 and mitochondrial complexes were co-eluted at fraction (27-30) in 293T cells. Dox, with doxycycline induction; no Dox, without doxycycline induction; TAM, tamoxifen. ATP5: ATP synthase 5; MTCO2: mitochondrially encoded cytochrome *c* oxidase II; NDUFA8: NADH: ubiquinone oxidoreductase subunit A8; PPOX: protoporphyrinogen oxidase.

k, Representative images of Mitotracker staining (Left) and quantification of mitochondria length (Right) of shCTL and sh*CHD6* HCT116 cells. Insets showing

larger magnifications of the boxed areas to visualize mitochondrial morphology. The irregular and shorter mitochondria length observed in *CHD6* KD cells.

Assays were performed with three replicates. p values were calculated by two-tailed t-test **(c, f, g)**, two-way ANOVA **(e)** or one-way ANOVA (**c, k**). Data are presented as mean ± SD. ***P<0.001, **P<0.01, *P<0.05. KD, knockdown

Supplementary Fig. S6 CHD6 regulates mitochondrial dynamic through TMEM65

a, Representative images of Mitotracker staining (Left) and quantification of mitochondria length (Right) of shCTL and sh*CHD6* HCT116 cells expressing TMEM65 or control vector. TMEM65 can reverse *CHD6* KD-mediated shorter mitochondria length. Data are presented as mean ± SD. **P<0.01, *P<0.05. p values were calculated by one-way ANOVA.

b, Representative images of Mitotracker staining in HCT116 cells transfected with GFP-TMEM65. Red, Mitotracker; Blue, DAPI; Green, GFP. TMEM65-GFP and mitochondria Mitotracker colocalized.

c, Representative transmission electron microscopy (TEM) images of HCT116 (Tet-on-sh*CHD6*) cells that were cultured in medium with or without doxycycline. Arrowheads indicate mitochondria**.** *CHD6* KD cells tended to have small mitochondria.

d, Immunoblot analysis of mitochondrial proteins in HCT116 (Tet-on-sh*CHD6*) cells that were cultured in medium with or without doxycycline. *CHD6* KD leads to increased Drp1 in the mitochondria.

e, Representative TEM images of HCT116 cells transfected with empty vector or TMEM65. Arrowheads indicate mitochondria. TMEM65 expression leads to longer fused mitochondria.

f, Mitochondrial protein levels of HCT116 cells transfected with empty vector or TMEM65. TMEM65 expression leads to the reduction of p-Drp1.

g, Representative TEM images showing the clear mitochondrial cristae in HCT116 cells with or without TMEM65 overexpression. Arrowheads indicate mitochondria. TMEM65 expression leads to more cristae per mitochondrion.

h, Immunoblot analysis of CHD6, TMEM65, PPOX, p-Drp1 and VDAC1 expression in colons obtained from the unchallenged *Chd6*$^{fl/fl}$ +TAM and *Chd6*$^{fl/fl}$/*Villin*CreER^{T} +TAM mice. PPOX: protoporphyrinogen oxidase; Drp1: dynamin-related protein 1; VDAC: Voltage-dependent anion-selective channels.

Supplementary Fig. S7 CHD6 interacts with TCF4 through ATPase domain

a, GSEA of Wnt pathway signature in CHD6 high verse low CRC samples.

b, GSEA of Wnt pathway signature in TMEM65 high verse low CRC samples.

c, GSEA of Wnt pathway signature in control verse CHD6 KD HCT116 cells. Normalized enrichment score (NES), Nominal P value of correlation were showed.

d, Schematic illustration of CHD6 domains and truncation mutations. The plus (+) and minus (-) signs indicate the positive and negative binding, respectively.

e, Co-IP of Myc-tagged TCF4 with Flag-tagged CHD6 WT or Flag-tagged CHD6 truncated mutants. The cell lysates were immunoprecipitated with an anti-Myc antibody and immunoblotted with indicated antibodies. WCL: whole cell lysates.

f, Schematic illustration of CHD6 domain deletions. The plus (+) and minus (-) signs indicate the positive and negative binding, respectively.

g, Co-IP of Myc-tagged TCF4 with Flag-tagged CHD6 WT or Flag-tagged CHD6 domain deletion mutants. The cell lysates were immunoprecipitated with an anti-Myc antibody and immunoblotted with indicated antibodies. WCL: whole cell lysates.

h, RT-qPCR analysis of *CHD6* in HCT116 cells treated with WNT pathway inhibitors IWR-1-endo (20 μM), XAV-939 (1 μM) or vehicle. IWR-1-endo and XAV939 downregulated *CHD6* expression.

i, RT-qPCR analysis of *CHD6* in HCT116 cells cultured with L-wnt3a cell conditioned medium (CM), in the presence or absence of WNT pathway inhibitors IWR-1-endo (20 μM) and XAV-939 (1 μM).

Assays were performed with three replicates. p values were calculated by one-way ANOVA (**h, i**). Data are presented as mean ± SD. ****P*<0.001, ***P*<0.01.

Supplementary Figure S1

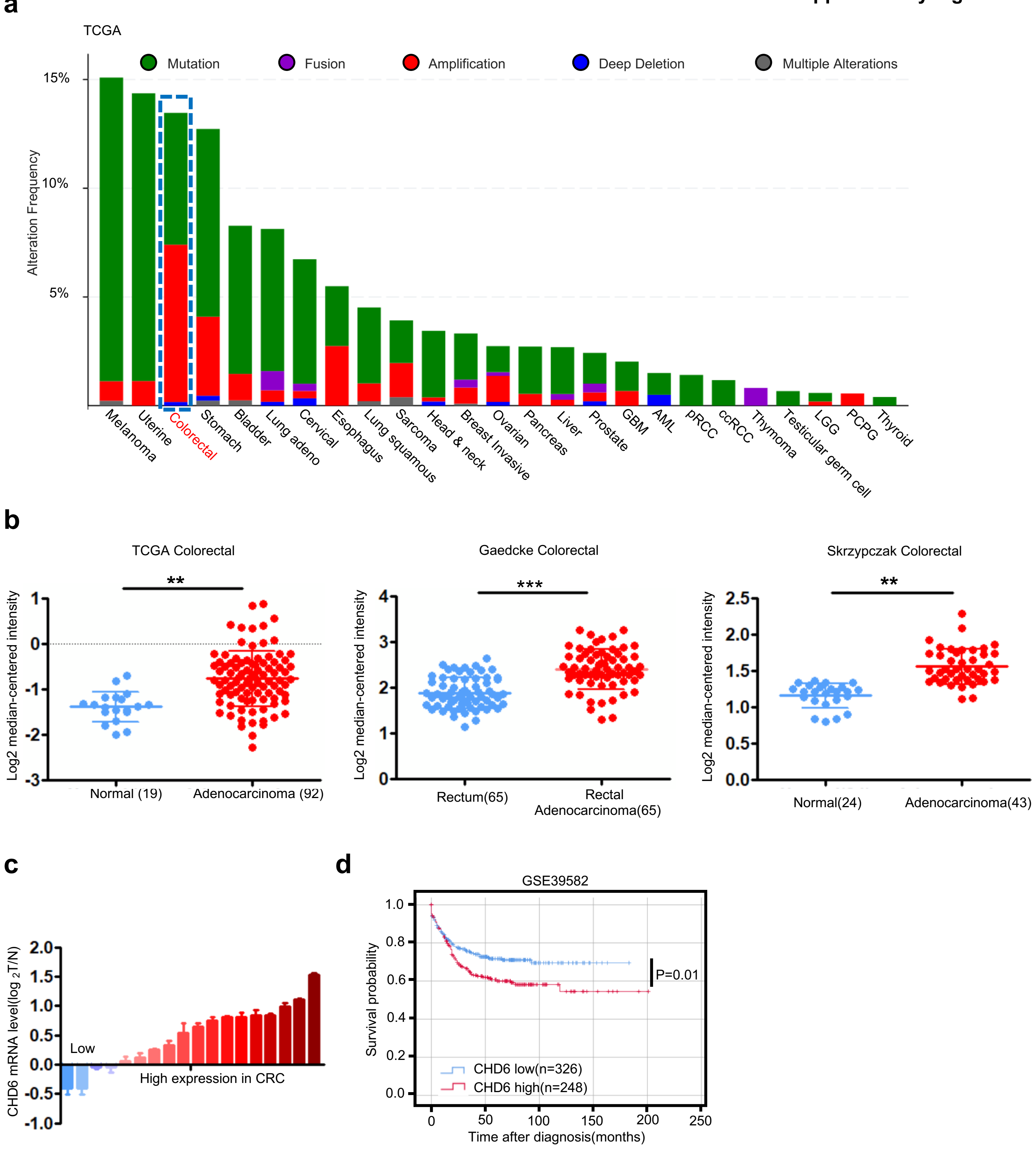

Supplementary Figure S2

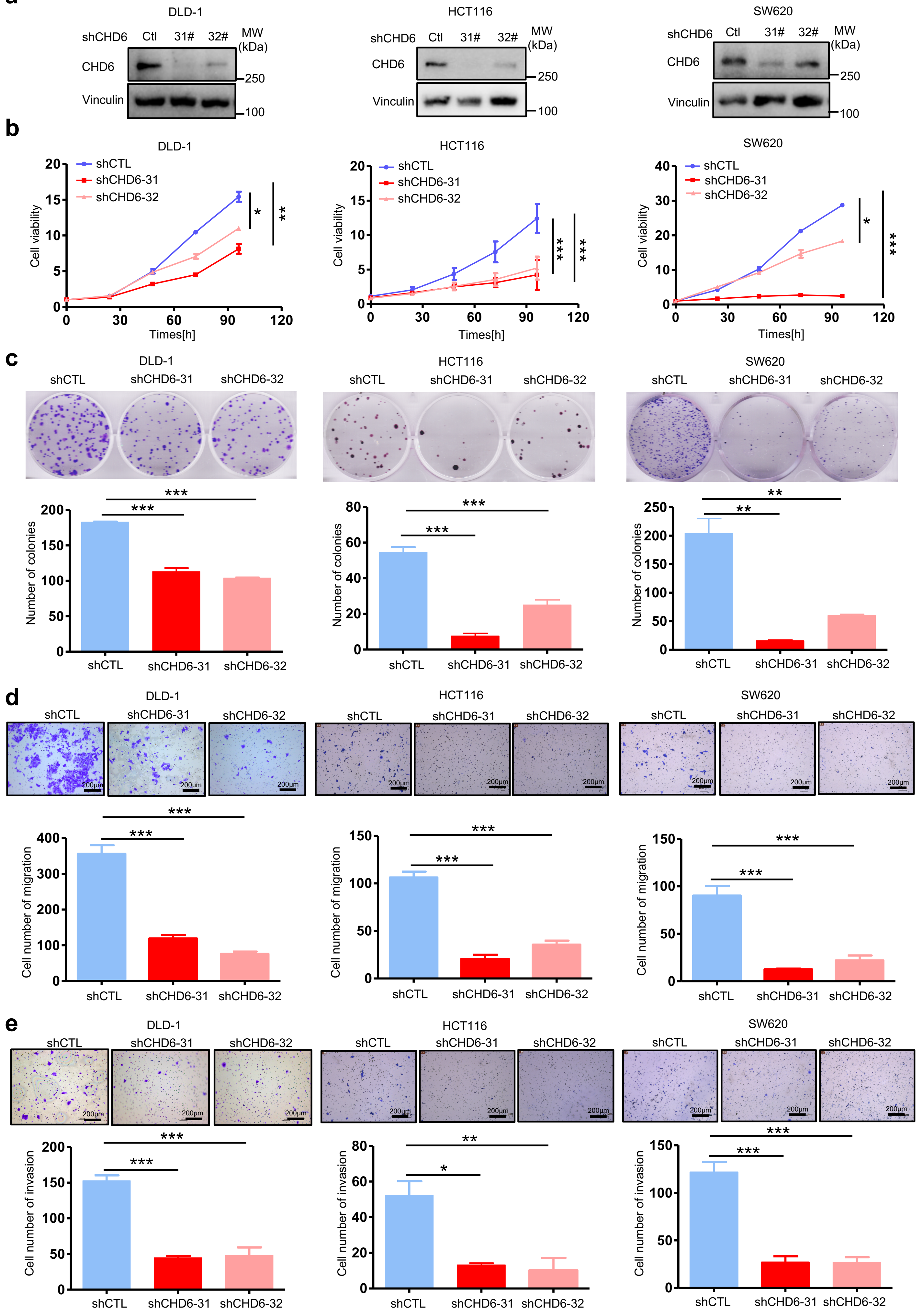

Supplementary Figure S3

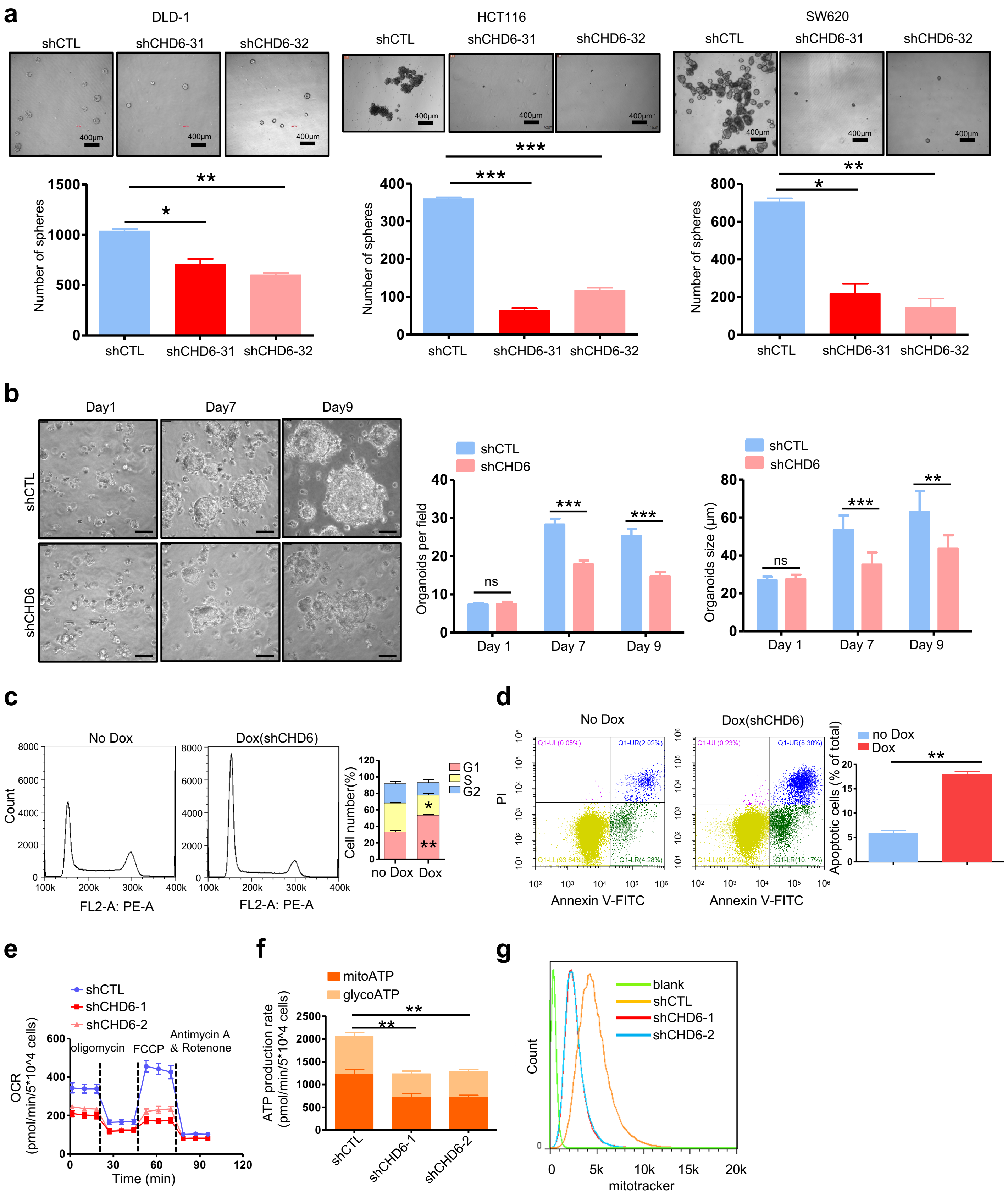

Supplementary Figure S4

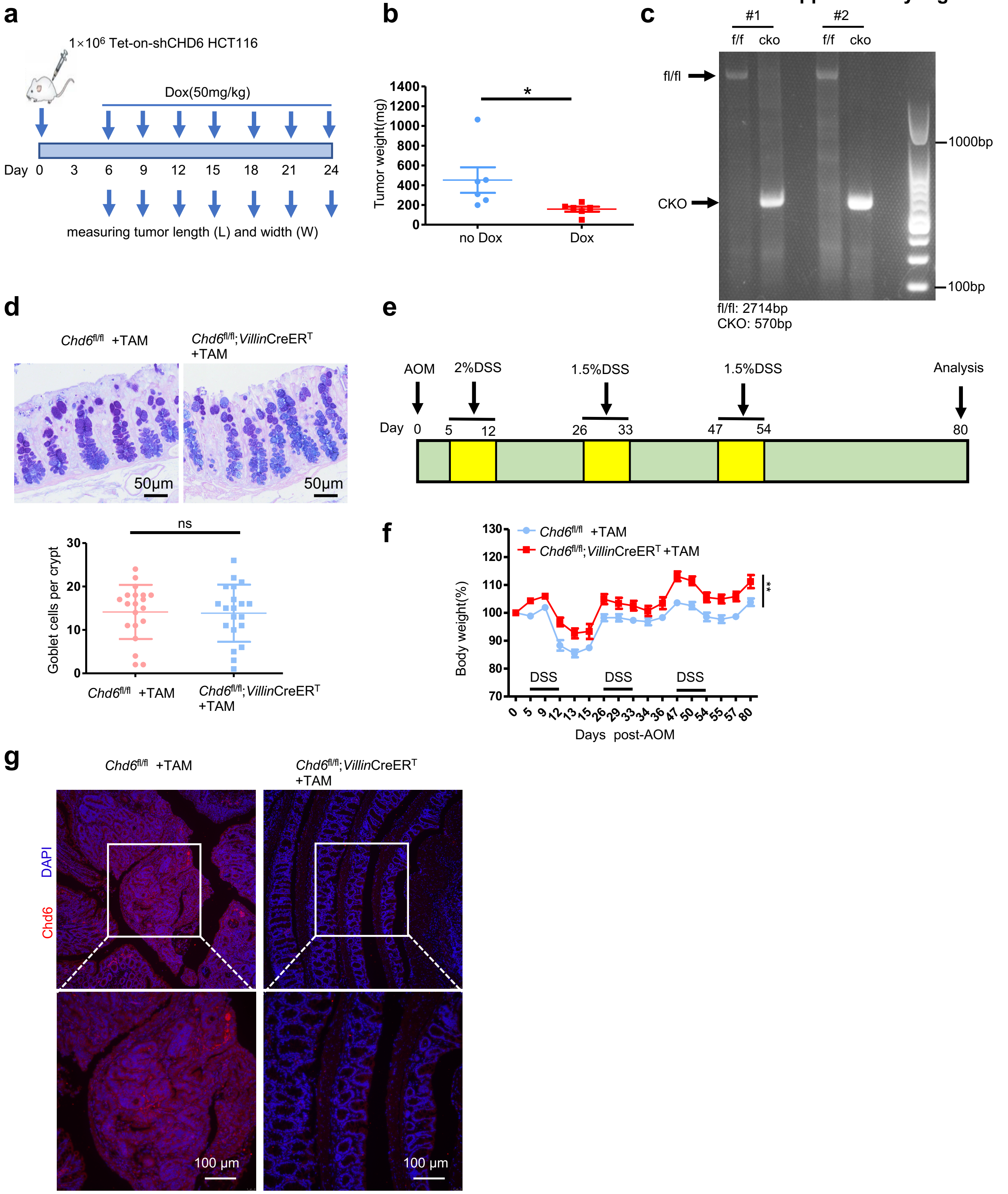

Supplementary Figure S5

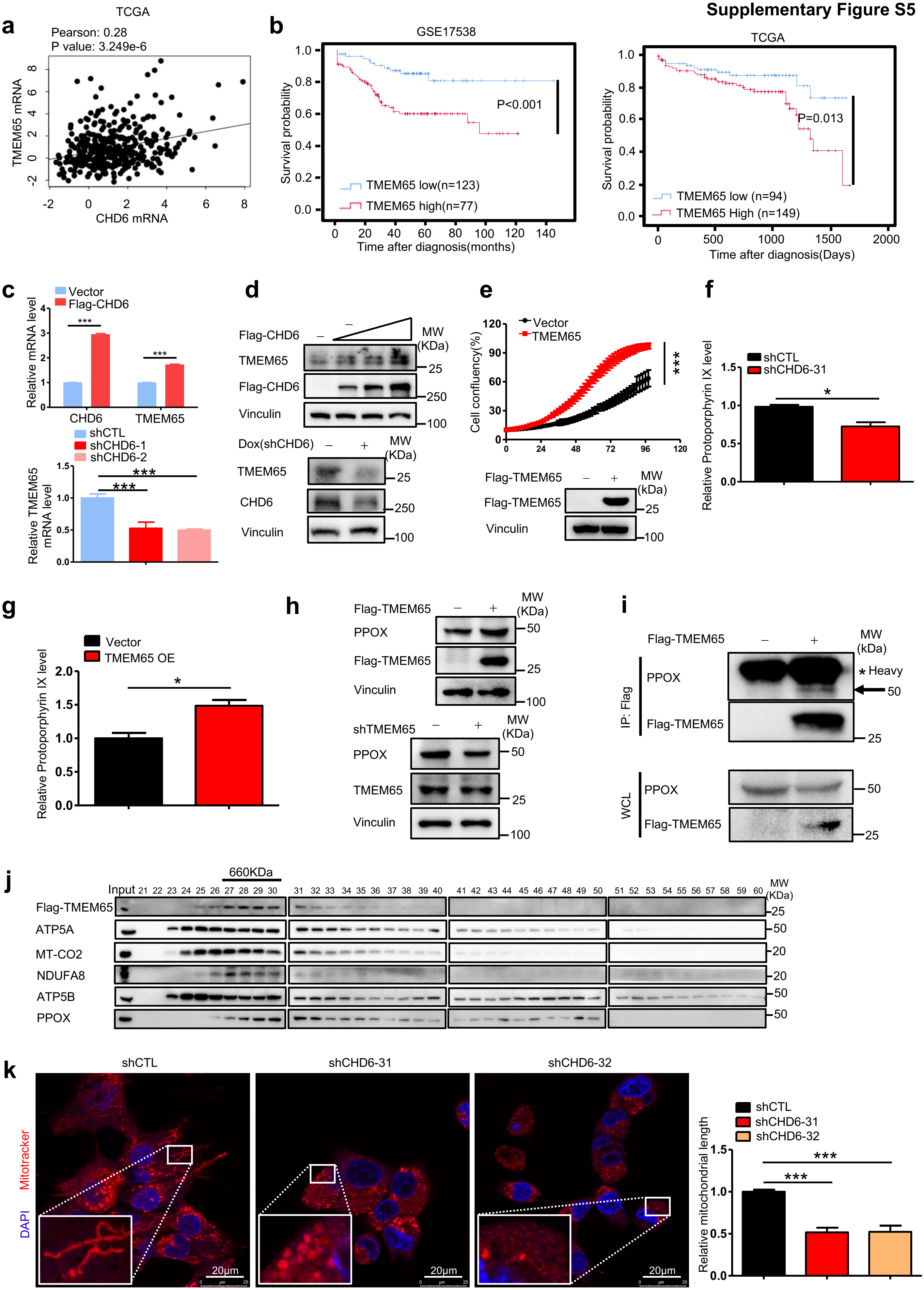

Supplementary Figure S6

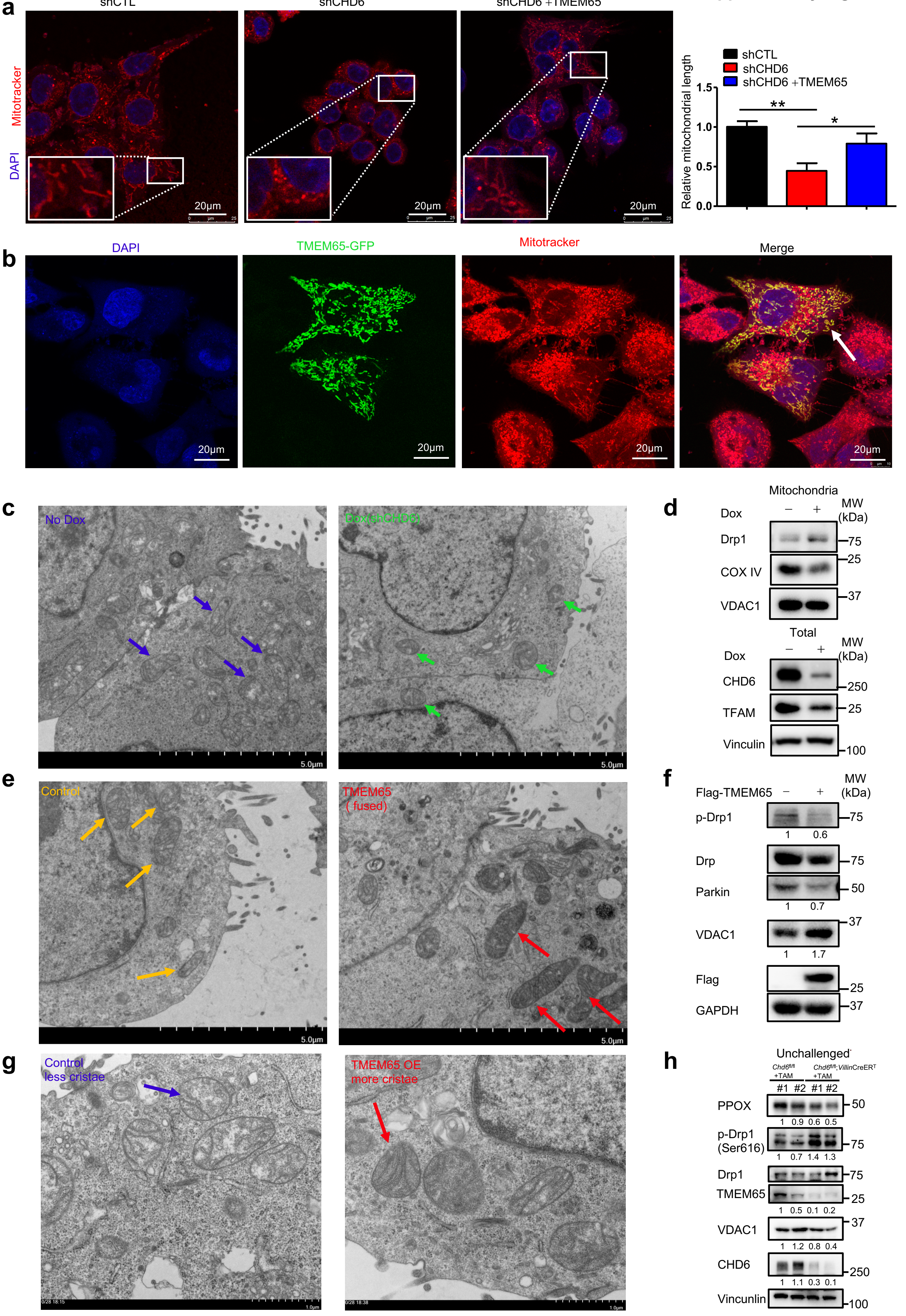

Supplementary Figure S7

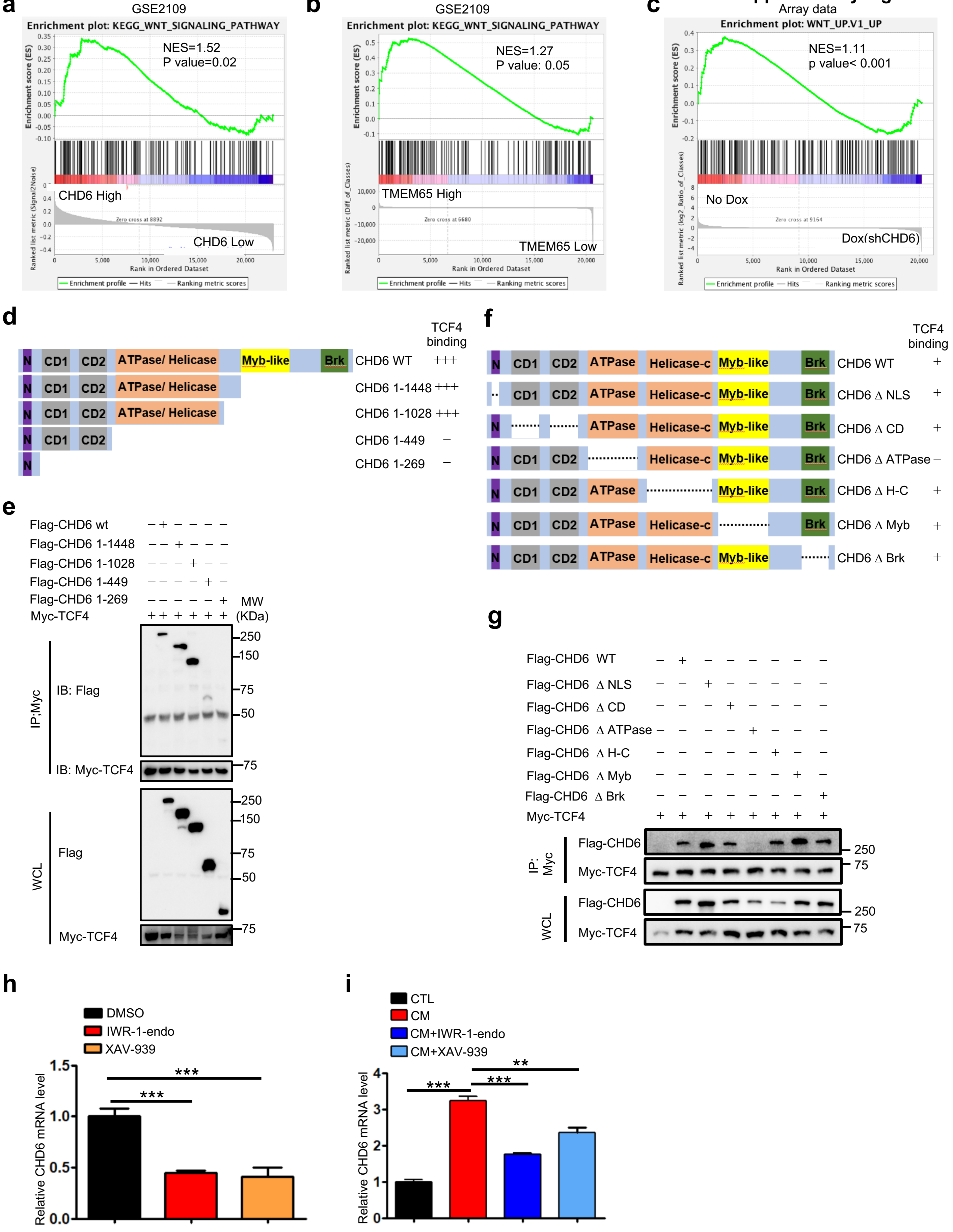

Table 1

Correlation between expression of CHD6 and TMEM65 and clinicopathological features of colorectal cancer patients

Variable	Low CHD6	High CHD6	p value[a]	Low TMEM65	High TMEM65	p value[a]
Gender			0.3725			0.4474
Male	22 (51.1)	36 (60.0)		19 (51.4)	39 (59.1)	
Female	21 (48.8)	24 (40.0)		18 (48.6)	27 (40.9)	
Median age			0.4068			0.4226
<59 years	10 (24.3)	10 (17.5)		6 (16.2)	14 (22.9)	
≥59 years	31 (75.6)	47 (82.4)		31 (83.8)	47 (77.1)	
Histological grade			0.3335			0.0646
I - II	2 (4.5)	4 (6.7)		2 (5.3)	4 (6.1)	
II	23 (52.3)	28 (47.5)		18 (47.4)	33 (50.8)	
II - III	11 (25.0)	22 (37.2)		9 (23.7)	24 (36.9)	
III	8 (18.1)	5 (8.4)		9 (23.7)	4 (6.2)	
pT status			0.4336			0.4900
T1	0 (0)	1 (1.7)		1 (2.7)	0 (0)	
T2	3 (7.1)	1 (1.7)		2 (5.4)	2 (3.2)	
T3	34 (80.9)	47 (81.0)		30 (81.1)	51 (81.0)	
T4	5 (11.9)	9 (15.5)		4 (10.8)	10 (15.9)	
pN status			0.0677			0.0570
N0	32 (76.2)	32 (54.2)		29 (78.4)	35 (56.4)	
N1	6 (14.2)	19 (32.2)		4 (10.8)	19 (30.6)	
N2	4 (9.5)	8 (13.6)		4 (10.8)	8 (12.9)	
pM status			0.7495			0.9069
M0	43 (97.7)	58 (96.6)		37 (97.4)	64 (97.0)	
M1	1 (2.3)	2 (3.3)		1 (2.6)	2 (3.0)	
Clinical stage			0.1902			0.0749
I	3 (6.8)	1 (1.7)		3 (7.9)	1 (1.5)	
II	29 (65.9)	31 (52.5)		26 (68.4)	34 (52.3)	
III	11 (25.0)	25 (42.4)		8 (21.1)	28 (43.1)	
IV	1 (2.2)	2 (3.4)		1 (2.6)	2 (3.1)	

p values were calculated in SPSS25 using a chi-square test. p values<0.05 were considered to indicate statistical significance.

Table 2

Univariate and multivariate analysis of different prognostic parameters for colorectal cancer patients

	Univariate analysis		Multivariate analysis	
**Variable**	**HR (95% CI)[a]**	**p value[b]**	**HR (95% CI)[a]**	**p value[b]**
cohort (n=104)				
Gender (male versus female)	0.9 (0.6-1.5)	0.730	0.7(0.4 -1.3)	0.300
Age (<59 years versus ≥59 years)	1.8 (0.9-3.7)	0.091	3.1(1.4-7.0)	0.006
Histological grade (I-II, II,II-III or III)	1.4 (1.0-1.9)	0.038	1.4 (0.9-2.0)	0.100
pT status (T1, T2, T3 or T4)	2.0 (1.2-3.5)	0.013	2.0 (1.1-3.9)	0.029
pN status (N0, N1 or N2)	2.3 (1.6-3.2)	<0.001	2.6 (1.6-4.0)	<0.001
pM status (M0 versus M1)	14.1 (4.0-50.3)	<0.001	8.7 (2.3-33.7)	0.002
CHD6 expression (low versus high)	2.8 (1.6-4.8)	<0.001	1.8 (0.9-3.5)	0.094
TMEM65 expression (low versus high)	3.0 (1.7-5.5)	<0.001	2.3 (1.1-4.7)	0.031

[a]Hazard ratios (HRs) and 95% confidence intervals (CIs) were calculated using univariate or multivariate Cox proportional hazards regression in SPSS 25.

[b]p values were calculated using univariate or multivariate Cox proportional hazards regression in SPSS 25. p values <0.05 were considered to indicate statistical significance.
